# Modifiable risk factors and skin cancers: a multi-omics Mendelian randomization study from causal inference to drug target discovery

**DOI:** 10.3389/fgene.2026.1870725

**Published:** 2026-07-07

**Authors:** Zhen Qin, Wuda Huoshen, Xueqing Li, Shiyu Li, Chen Sun, Sha Yi

**Affiliations:** 1 Department of Rheumatology and Immunology, The Affiliated Hospital, Southwest Medical University, Luzhou, Sichuan, China; 2 School of Stomatology, Southwest Medical University, Luzhou, Sichuan, China; 3 Department of Periodontics and Oral Mucosal Diseases, The Affiliated Stomatology Hospital, Southwest Medical University, Luzhou, Sichuan, China; 4 Department of Dermatology, Chengdu Integrated TCM & Western Medicine Hospital, Chengdu, China

**Keywords:** basal cell carcinoma, melanoma, Mendelian randomization, risk factors, squamous cell carcinoma

## Abstract

**Introduction:**

Recent studies have linked modifiable risk factors (RFs) to melanoma, basal cell carcinoma (BCC), and squamous cell carcinoma (SCC). This study aimed to investigate the causal relationships between 11 modifiable RFs and these skin cancers and to identify novel therapeutic targets using multi-omics approaches.

**Methods:**

Exposure data were obtained from genome-wide association studies (GWAS). Mendelian randomization (MR) analyses were performed using the inverse variance weighted (IVW) method as the primary approach, and results from discovery and replication cohorts were combined by meta-analysis. Functional Mapping and Annotation (FUMA) and summary-data-based MR (SMR) were used to prioritize therapeutic targets. Drug prediction, phenome-wide association studies (PheWAS), and single-cell analyses were conducted to evaluate target druggability and biological relevance.

**Results:**

Actinic keratosis (AK) was associated with an increased risk of melanoma (OR = 1.24, 95% CI 1.07-1.43, P < 0.01), whereas alcohol consumption was negatively associated with SCC risk (OR = 0.77, 95% CI 0.62-0.95, P = 0.02). No causal relationships were observed between the investigated RFs and BCC. One potential therapeutic target for melanoma (EDEM2, PSMR = 0.03) and five candidate therapeutic targets for SCC (MAPK3, PSMR = 5.30E-04; NRBP1, PSMR = 4.32E-04; ANKK1, PSMR = 1.89E-06; IL27, PSMR = 3.04E-03; ADH5, PSMR = 0.02) were identified. Drug prediction, PheWAS, and single-cell analyses further supported the therapeutic potential of these genes.

**Discussion:**

AK appears to increase the risk of melanoma, whereas alcohol consumption may be protective against SCC. EDEM2 may represent a potential therapeutic target for melanoma, while MAPK3, NRBP1, ANKK1, IL27, and ADH5 are promising candidate targets for SCC. Further experimental and clinical studies are warranted to validate these findings.

## Introduction

Cutaneous malignancies, including melanoma, basal cell carcinoma (BCC), and squamous cell carcinoma (SCC), represent the most common cancers worldwide (Perez et al., 2022; Sung et al., 2021; Perera et al., 2015; Linares et al., 2015). Melanoma causes most skin cancer deaths despite fewer cases (Sung et al., 2021). Rising incidence demands better prevention and treatment (Aggarwal et al., 2021; Khan et al., 2022). Melanoma pathogenesis involves complex aberrations in multiple signaling pathways, including CDK4/CDKN2A, MAPK, and BRAF, contributing to the limited efficacy of current targeted therapies (Hauschild et al., 2012; Ascierto et al., 2013; Sosman et al., 2012; Tod et al., 2020; Mao et al., 2021). Similarly, while PD-1 antibodies show promise for SCC, and SMO inhibitors such as sonidegib and vismodegib have proven effective against BCC, treatment resistance and moderate response rates remain significant hurdles (Miao et al., 2019; Amôr et al., 2021; Chmiel et al., 2022). Thus, new therapeutic targets are needed.

Prevention strategies for melanoma and keratinocyte carcinomas (KCs) primarily target modifiable risk factors (RFs), including smoking, alcohol use, serum vitamin D levels, HIV infection, actinic keratosis (AK), nonionizing radiation, sunburns, and ultraviolet (UV) radiation exposure (Perez et al., 2022). Among these, UV exposure is the most established carcinogen, inducing DNA mutations and sunburn (Lee et al., 2020; Wood et al., 2023). While associations between alcohol consumption and BCC risk, as well as smoking and SCC risk, have been reported, the influence of daily lifestyles on cutaneous cancers remains controversial (Wu et al., 2015; Leonardi-Bee et al., 2012; Pirie et al., 2018; Asgari et al., 2017; Sawada and Nakamura, 2021). Despite these established links, research on therapeutic targets derived from modifiable RFs is limited. Investigating the molecular pathways influenced by these factors may uncover novel intervention strategies for skin cancer.

Mendelian randomization (MR) uses genetic variants as instrumental variables to infer causal relationships between exposures and diseases, minimizing confounding and reverse causation (Davey Smith and Hemani, 2014; Burgess et al., 2017). Expression quantitative trait locus (eQTL) mapping reveals the impact of genetic variants on gene expression (Albert and Kruglyak, 2015). Cis-eQTLs, located near drug target genes, are particularly useful for prioritizing potential therapeutic targets (Zhu et al., 2016; Pavlides et al., 2016). Here, we employed MR to assess causal links between modifiable RFs and skin cancers. We then performed summary-data-based MR (SMR) integrating eQTL, methylation QTL (mQTL), and protein QTL (pQTL) data to identify druggable genes and understand their regulatory architecture. Additionally, phenome-wide association (PheWAS) analysis and single-cell expression profiling were conducted to assess the therapeutic potential and cell-type specificity of candidate targets.

## Methods

### Study design and data sources

**FIGURE 1:**
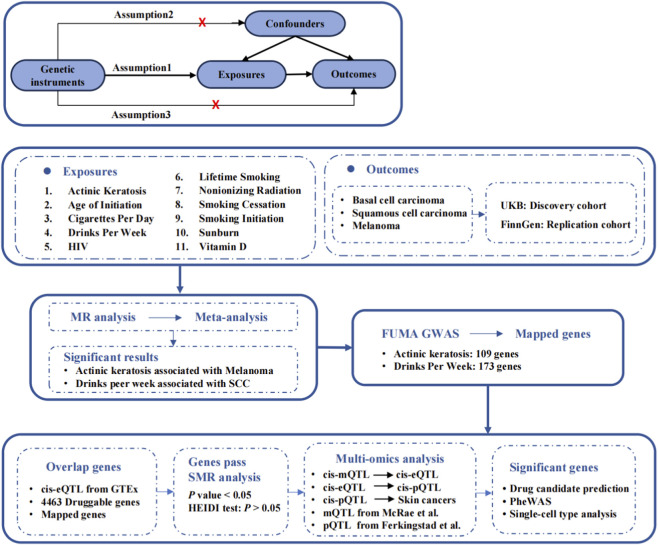
The study design and flowchart in our analysis. UKB, United Kingdom Biobank; QTL, quantitative trait loci; GTEx, Genotype-Tissue Expression.

The study design is shown in Figure 1. An overall MR analysis was conducted to assess causal relationships between 11 modifiable risk factors (RFs) and three skin cancers. This study followed three basic MR hypotheses: (i) SNPs are strongly associated with the RFs; (ii) SNPs are independent of known confounders (including carcinogens, age, PM2.5, viruses, and immune factors); (iii) SNPs influence these three skin cancers exclusively through their effects on the RFs.

### Exposure data sources

Genetic instruments for 11 modifiable risk factors were obtained from public GWAS datasets. Smoking and alcohol traits came from GSCAN (Seviiri et al., 2022): alcohol consumption (N = 941,280), smoking initiation (N = 1,232,091), cigarettes per day (N = 337,334), and smoking cessation (N = 547,219). Lifetime smoking and sunburn data were from UKB (Loh et al., 2018; Wootton et al., 2020), with 462,690 and 350,232 Europeans, respectively. AK and nonionizing radiation instruments (both N = 361,194) came from IEU Open GWAS (https://gwas.mrcieu.ac.uk/datasets/) (Neale lab). Vitamin D data were from a meta-analysis of multiple cohorts (Zheng et al., 2020): EPIC-InterAct (N = 18,078), EPIC-Norfolk (N = 10,231), EPIC-CVD (N = 12,253), Ely (N = 690), and SUNLIGHT (N = 79,366). HIV data (N = 218,792) were from FinnGen.

## Outcome data source

Outcome data were obtained from large-scale GWAS summary statistics of three major skin cancers. For the discovery analyses, basal cell carcinoma (BCC) and squamous cell carcinoma (SCC) GWAS data were derived from the multi-phenotype GWAS conducted by Seviiri et al. (2022), which included 2,523,630 participants for BCC and 2,529,399 participants for SCC, primarily of European ancestry from the United Kingdom Biobank and related cohorts. Melanoma GWAS summary statistics were obtained from the United Kingdom Biobank (ieu-b-4969; N = 375,767), comprising individuals of European ancestry.

For replication analyses, independent GWAS summary statistics were obtained from the FinnGen consortium. The BCC dataset included 334,699 participants (20,506 cases and 314,193 controls), the SCC dataset included 317,724 participants (3,531 cases and 314,193 controls), and the melanoma dataset included 317,387 participants (3,194 cases and 314,193 controls). Detailed information regarding the outcome datasets is provided in Supplementary Table S1.

### Instrumental variable selections

Instrumental variables (IVs) were selected based on three criteria: (i) *P*-value thresholds (AK, HIV, nonionizing radiation: *P* < 1 × 10^−5^; other eight RFs: *P* < 5 × 10^−8^); (ii) LD clumping (*r* ^*2*^ < 0.001, window = 10,000 kb) to ensure independence; (iii) exclusion of confounder-associated SNPs using Phenoscanner (http://www.phenoscanner.medschl.cam.ac.uk) (Staley et al., 2016; Lin et al., 2025b). Genetic variants associated with each exposure were selected as instrumental variables using a genome-wide significance threshold of *P* < 5 × 10^−8^. For actinic keratosis and HIV infection, however, the number of SNPs reaching this threshold in the available GWAS datasets was insufficient to construct valid instruments. Therefore, a more relaxed significance threshold (*P* < 1 × 10^−5^) was applied for these two exposures to retain an adequate number of instrumental variables and ensure sufficient statistical power for Mendelian randomization analyses. This approach has been widely adopted in previous MR studies when investigating traits with limited genome-wide significant genetic variants. All selected instrumental variables had F-statistics >10, suggesting that weak instrument bias was unlikely to materially influence the results.

### Mendelian randomization

Five MR methods estimated causal effects: inverse-variance weighted (IVW), MR Egger, weighted median, weighted mode, and simple mode. IVW was the primary analysis, combining Wald ratios via meta-analysis (Bowden et al., 2016; Lin et al., 2024). MR Egger detected directional pleiotropy (intercept indicates magnitude). Weighted median/mode provide consistent estimates when ≤50% of weight is invalid. Simple mode was a complementary method. Results are odds ratios (ORs) with 95% CIs.

### Sensitivity analysis

Sensitivity analyses included Cochran’s *Q* test (heterogeneity), MR Egger intercept test (directional pleiotropy), and MR-PRESSO (outlier detection/correction with 1,000 distributions) (Verbanck et al., 2018; Wu et al., 2020). Leave-one-out analysis assessed individual SNP influence. Funnel and scatter plots visualized heterogeneity and pleiotropy.

### Meta-analysis

Meta-analysis synthesized causal estimates from discovery and replication cohorts (Niu and Gu, 2016). For results with significant heterogeneity or pleiotropy, MR-PRESSO-corrected estimates were used. Estimates with persistent pleiotropy were excluded (Xie et al., 2023). Fixed-effects models were applied for low heterogeneity (*I* ^*2*^ ≤ 50%); random-effects for substantial heterogeneity (*I* ^*2*^ > 50%) (Morze et al., 2022). If only one reliable estimate was available, the causal relationship was considered inconclusive and excluded.

### Druggable gene prioritization

#### Functional Mapping and Annotation (FUMA) of GWAS for mapped genes

The FUMA pipeline (v1.3.5d) annotated GWAS results and mapped SNPs to genes using positional and functional information (Watanabe et al., 2017). SNP2GENE (default settings) identified risk loci with *P*-value thresholds (AK: *P* < 1 × 10^−5^; drinks/week: *P* < 5 × 10^−8^) and LD filtering (*r* ^*2*^ < 0.6). Lead SNPs were independent associations (LD *r* ^*2*^ < 0.1) within 250 kb. LD reference data came from 1,000 Genomes Phase 3 (all ancestries) (Auton et al., 2015; Watanabe et al., 2017).

### SMR analysis

Summary-data-based MR (SMR) integrates GWAS and eQTL data with higher statistical power than conventional MR by leveraging top-associated cis-QTLs (Zhou et al., 2020). To identify therapeutic targets for BCC patients with AK and SCC patients with alcohol consumption, we used a previously established list of 4,463 druggable genes (Finan et al., 2017; Lin et al., 2025a). Whole blood cis-eQTL (cis-expression quantitative trait loci) data were obtained from GTEx v8 (https://gtexportal.org/home/), comprising 838 donors across 52 tissues and two cell lines (Albert and Kruglyak, 2015). The top cis-eQTLs within ±1,000 kb of each gene was selected at *P* < 5 × 10^−8^. SNPs with allele frequency differences >0.2 between LD reference, eQTL, and outcome datasets were excluded. Mapped genes from FUMA were matched with druggable genes and GTEx cis-eQTLs for SMR analysis, followed by HEIDI test to distinguish pleiotropy from linkage. Associations with *P* < 0.05 and *P*-HEIDI >0.05 were considered significant.

### Integrating evidence from multi-omic level

To understand multi-level regulatory relationships between drug targets and skin cancers, we performed SMR using mQTL (N = 1,980) and eQTL data for upstream regulation, and eQTL and pQTL (N = 35,559) data for downstream regulation (McRae et al., 2018; Ferkingstad et al., 2021). As proteins are the ultimate functional products of genes, establishing protein-level causality is critical; thus, SMR was conducted to refine associations between target-related proteins and skin cancers.

### Candidate drug prediction

To assess whether prioritized genes are viable drug targets, we predicted candidate drugs using Enrichr (DSigDB, http://amp.pharm.mssm.edu/Enrichr) (Kuleshov et al., 2016). Genes significant in SMR analysis were uploaded to the platform, which hosts 180,184 gene sets from 102 libraries, to identify potential therapeutic compounds targeting these genes.

### Phenome-wide association analysis

To evaluate horizontal pleiotropy and potential side effects of prioritized drug targets, we performed PheWAS using the AstraZeneca PheWAS Portal (https://azphewas.com/) (Wang et al., 2021). This portal includes ∼1,500 phenotypes from United Kingdom Biobank (N ≈ 450,000). A significance threshold of 2 × 10^−9^ (portal default) minimized false positives.

### Single cell sequence analysis

Single-cell RNA-seq data from SCC (GSE20334) and melanoma (GSE72056) were analyzed for cell type-specific gene expression (Ferlini et al., 2010; Tirosh et al., 2016). The Seurat pipeline was used for (Butler et al., 2018): performed: 1. data conversion to Seurat objects. 2. quality control (excluding low-quality cells by mitochondrial/ribosomal genes). 3. identification of top 2,000 variable genes (FindVariableFeatures). 4. gene correlation analysis for quality assessment. 5. dimensionality reduction (PCA and UMAP) (Becht et al., 2018). TPM normalization and scaling. 7. cell type annotation (SingleR) (Aran et al., 2019).

## Statistics

All analyses were performed in R v4.3.1 (R Foundation for Statistical Computing, Vienna, Austria) with packages: TwoSample MR, Seurat, forestplot, SingleR, and meta. IVW was the primary MR method (Lin et al., 2021), random-effects IVW replaced fixed-effects IVW when heterogeneity persisted after multiple testing correctio (Li et al., 2023a). Multiple testing correction was not applied to allow exploratory analysis. Statistical significance was set at *P* < 0.05.

## Results

### MR analysis between modifiable RFs and skin cancers

Cohort details and instrumental variables are in Supplementary Tables S1–S12 (all *F* > 10). IVW results before pleiotropy removal are in Supplementary Figure S1. Full MR and sensitivity results for melanoma, BCC, and SCC are in Supplementary Tables S13, S14.

### The combined results of skin cancers risk from the meta-analysis

Meta-analysis results are in Supplementary Figures S2–S34. For BCC, drinks per week and vitamin D were excluded due to pleiotropy; no significant associations remained (Supplementary Figure S35). For SCC, drinks per week was negatively associated with risk in UKB (*OR* = 0.764, 95% *CI* 0.610–0.957, *P* = 0.019) and after meta-analysis (*OR* = 0.77, 95% *CI* 0.62–0.95, *P* = 0.02) (Supplementary Figure S36). For melanoma, AK was significant in UKB (*OR* = 1.238, 95% *CI* 1.071–1.431, *P* = 0.004) and after meta-analysis (*OR* = 1.24, 95% *CI* 1.07–1.43, *P* < 0.01), but not in FinnGen (Supplementary Figure S37). No other exposures were significant.

### FUMA GWAS

FUMA identified 173 genes for drinks per week and 109 for AK (Supplementary Tables S15, S16), with 62 and 59 leading SNPs, respectively (Supplementary Tables S17, S18). SNP functional consequences and per-locus summaries are in Supplementary Figures S38–S41. MAGMA results are in Supplementary Figures S32–S43.

### SMR analysis

**FIGURE 2:**
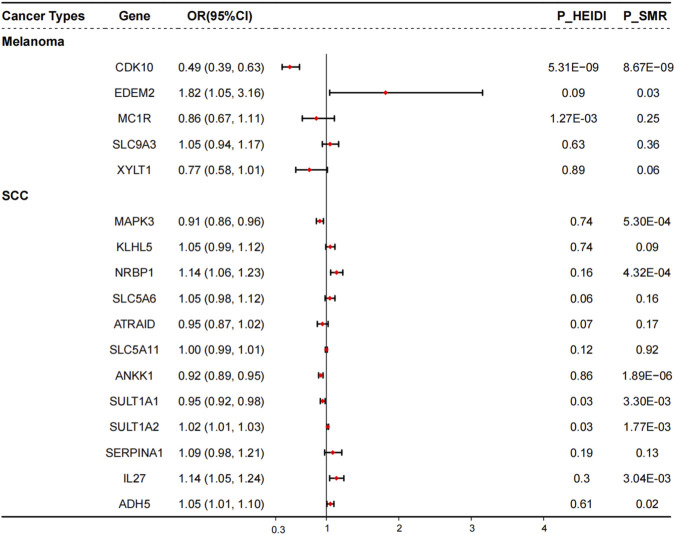
The SMR results between cis-eQTLs of RFs on skin cancers. OR odds ratio; CI confidence interval; SMR, summary-data-based Mendelian randomization. *EDEM2* was associated with the risk of melanoma; *MAPK3* and *ANKK1* were associated with the protection of SCC; *NRBP*, *1IL27* and *ADH5* and with the risk of SCC.

**FIGURE 3:**
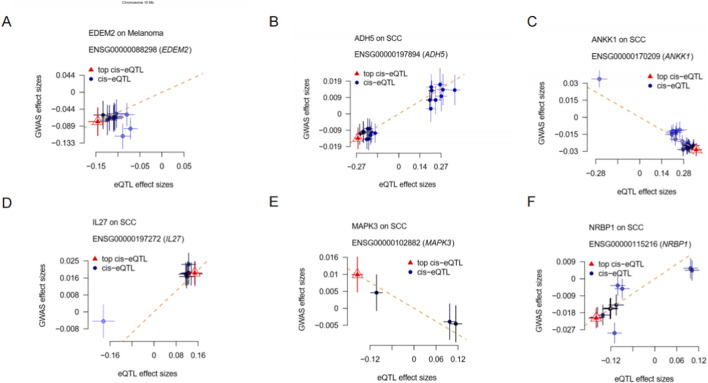
The SMR effect plot of significant eQTLs on skin cancers. **(A)** SMR effect plot of *EDEM2*; **(B)** SMR effect plot of *ADH5*; **(C)** SMR effect plot of *ANKK1*; **(D)** SMR effect plot of *IL*27; **(E)** SMR effect plot of *MAPK3*; **(F)** SMR effect plot of *NRBP1*.

**FIGURE 4:**
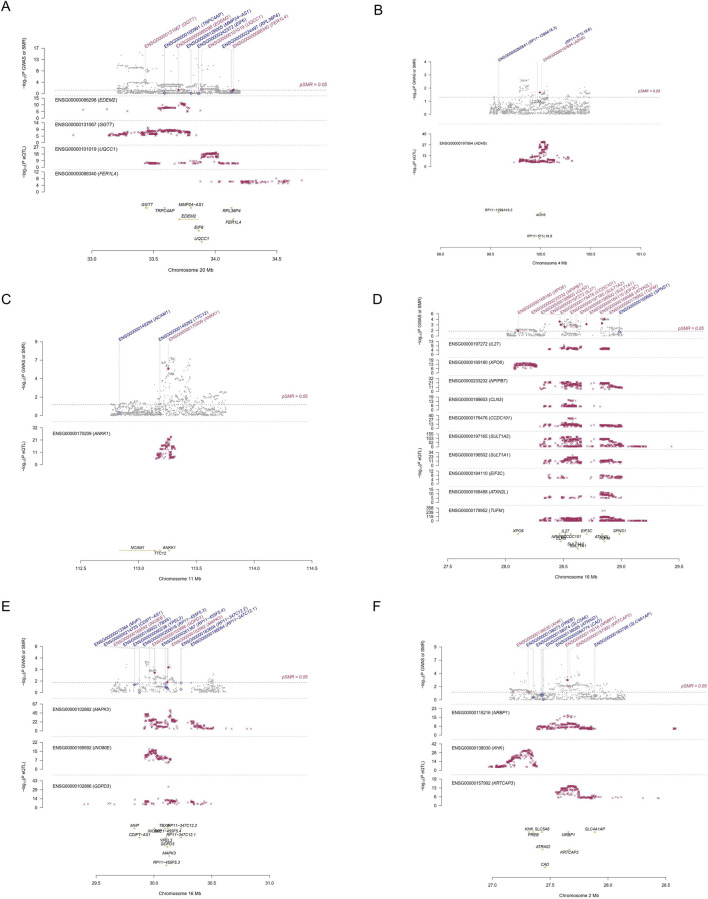
Regional SMR association plots for prioritized melanoma and SCC target genes. **(A)** SMR locus plot of *EDEM2*; **(B)** SMR locus plot of *ADH5*; **(C)** SMR locus plot of *ANKK1*; **(D)** SMR locus plot of *IL27*; **(E)** SMR locus plot of *MAPK3*; **(F)** SMR locus plot of NRBP1. The upper panels display GWAS and SMR association signals across each genomic locus, while the lower panels show the corresponding cis-eQTL association signals. Blue labels denote the prioritized candidate genes selected for downstream analyses, whereas red labels indicate neighboring genes located within the same genomic region for locus annotation. The figure highlights the genomic regions containing the prioritized targets *EDEM2, ADH5, ANKK1, IL27, MAPK3*, and *NRBP1*. The red dashed line represents the SMR significance threshold.

To prioritize potential therapeutic targets for melanoma, we cross-referenced the candidate risk genes mapped via FUMA with the druggable gene database and whole-blood cis-eQTL datasets from GTEx v8. This integration identified 5 candidate cis-eQTLs that genetically intersected with both actinic keratosis (AK) pathways and melanoma risk. Subsequent SMR analysis revealed that 1 gene, *EDEM2* (*P*_SMR_ = 0.03, *P*_HEIDI_ = 0.09), reached statistical significance, providing a robust genetic link between target-tissue expression regulation and melanoma (Figure 2). 12 cis-eQTLs were matched in SCC with drinks per week, and 5 genes were significant in SMR analysis (*MAPK3*: *P*_SMR_ = 5.30E-04, *P*_HEIDI_ = 0.74; *NRBP1*: *P*_SMR_ = 4.32E-04, *P*_HEIDI_ = 0.16; *ANKK1*: *P*_SMR_ = 1.89E-06, *P*_HEIDI_ = 0.86; *IL27*: *P*_SMR_ = 3.04E-03, *P*_HEIDI_ = 0.30; *ADH5*: *P*_SMR_ = 0.02, *P*_HEIDI_ = 0.61) in Figure 2. The effect plots of significant eQTLs on skin cancers are shown in Figure 3, while the SMR locus plots of significant eQTLs on skin cancers are shown in Figure 4.

### Multi-omic level integrating

**FIGURE 5:**
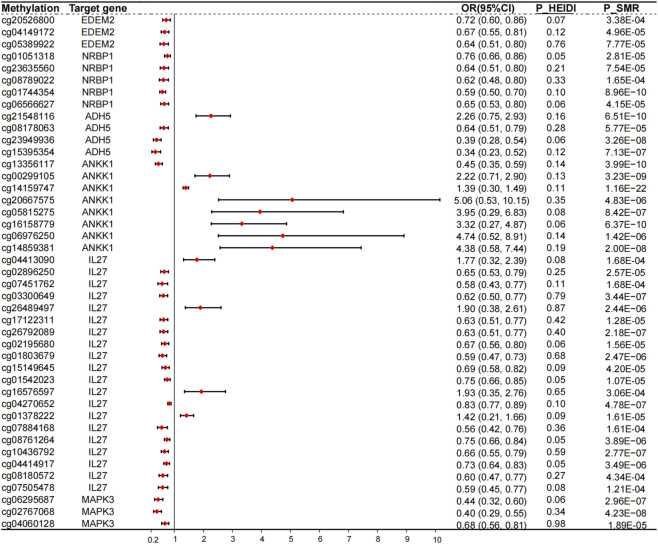
The forest plot of SMR result between cis-mQTL and cis-eQTL. In melanoma, three CpG sites (cg20526800, cg04149172, and cg05389922) were negatively associated with EDEM2 expression. In SCC, methylation sites showed significant associations with several identified drug targets. Five CpG sites were associated with NRBP1. For ADH5, cg21548116 was positively associated, whereas cg08178063, cg23949936, and cg15395354 were negatively associated. For ANKK1, cg13356117 was negatively associated, while seven CpG sites (cg00299105, cg14159747, cg20667575, cg05815275, cg16158779, cg06976250, and cg14859381) showed positive associations. Three CpG sites (cg04413090, cg26489497, and cg16576597) were positively associated with IL27, whereas seventeen CpG sites exhibited negative associations. Additionally, three CpG sites (cg06295687, cg02767068, and cg04060128) were negatively associated with MAPK3. Positive and negative associations are indicated by effect estimates greater than or less than zero, respectively.

In the causal effects of methylation on the melanoma drug target EDEM2, three sites (cg20526800, cg04149172, and cg05389922) were found to be negatively associated with *EDEM2* expression (Figure 5). In the SCC, various methylation sites revealed associations with identified drug targets (Figure 5). For instance, five sites (cg01051318, cg23635560, cg08789022, cg01744354, cg06566627) were negatively associated with *NRBP1*, while cg21548116 was positively associated with *ADH5*. Conversely, three sites (cg08178063, cg23949936, cg15395354) were negatively associated with *ADH5*. Additionally, cg13356117 was negatively associated with *ANKK1*, while seven sites (cg00299105, cg14159747, cg20667575, cg05815275, cg16158779, cg06976250, cg14859381) were positively associated with *ANKK1*. Furthermore, three sites (cg04413090, cg26489497, cg16576597) were positively associated with *IL27*, whereas 17 sites (cg02896250, cg07451762, cg03300649, cg17122311, cg26792089, cg02195680, cg01803679, cg15149645, cg01542023, cg04270652, cg01378222, cg07884168, cg08761264, cg10436792, cg04414917, cg08180572, cg07505478) were negatively associated with *IL27*. Finally, three sites (cg06295687, cg02767068, cg04060128) were negatively associated with *MAPK3*.

**FIGURE 6:**
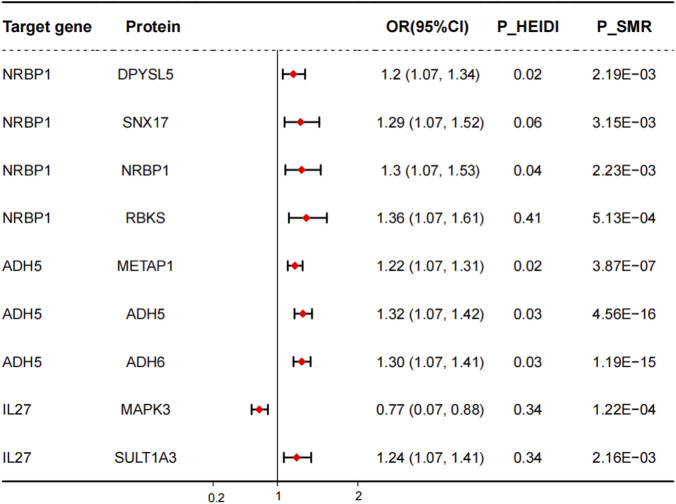
The forest plot of SMR result between cis-eQTL and cis-pQTL. SMR analysis integrating cis-eQTL and cis-pQTL datasets identified downstream proteins associated with prioritized SCC drug targets. Positive and negative effect estimates are shown with corresponding 95% confidence intervals.

To further elucidate the downstream molecular cascades driving squamous cell carcinogenesis, we performed an exploratory SMR analysis integrating whole-blood cis-eQTL signals with cis-pQTL data to evaluate target protein abundance changes (P_HEIDI >0.01). Within this regulatory chain, the cis-eQTLs of *NRBP1* were found to be positively associated with the levels of four downstream proteins (DPYSL5, SNX17, NRBP1, and RBKS), whereas the cis-eQTLs of *ADH5* were positively associated with three proteins (METAP1, ADH6, and ADH5) (Figure 6). In contrast, no downstream proteins passed the HEIDI test for the melanoma target *EDEM2*. Furthermore, the cis-eQTLs of *IL27* exhibited a negative causal association with MAPK3 protein levels but a positive correlation with SULT1A3 protein expression (Figure 6).

**FIGURE 7:**
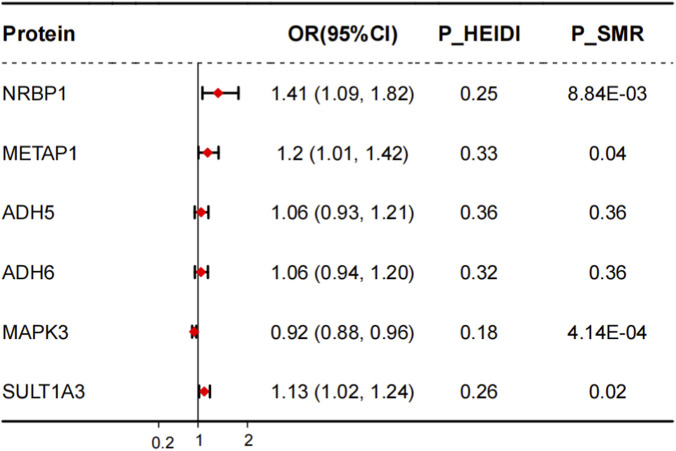
The forest plot of SMR result between cis-pQTL and SCC. SMR analysis using cis-pQTL instruments was performed to evaluate the causal effects of downstream protein expression on SCC risk. Genetically predicted increases in NRBP1, METAP1, and SULT1A3 protein levels were associated with increased SCC risk, whereas higher MAPK3 protein expression showed a protective effect. Effect estimates are presented with 95% confidence intervals, and associations passing the HEIDI test *P*_HEIDI_ > 0.01) are displayed.

To close the multi-omic loop, we subsequently utilized these cis-pQTL instruments to evaluate the direct causal effects of protein expression on SCC risk. This integration confirmed that genetically predicted increases in NRBP1, METAP1, and SULT1A3 protein levels were significantly linked to an elevated risk of SCC, whereas increased MAPK3 protein expression demonstrated a robust protective effect against SCC development (Figure 7). This progressive cis-eQTL-to-cis-pQTL-to-phenotype cascade establishes a rigorous genetic framework anchoring our prioritized targets to functional proteomic outcomes.

### Drug prediction and PheWAS

DSigDB predicted top 10 drug candidates (Table 1). For SCC (negatively associated with drinks per week), IL27 and MAPK3 interacted with multiple compounds (full list in Supplementary Tables S19, S20). For melanoma (positively associated with AK), EDEM2 interacted with ciclosporin and thapsigargin. Full results in Supplementary Tables S19, S20.

**TABLE 1:** Identification of the top 10 potential chemical compounds targeting prioritized risk genes in skin cancers.

Cancer	Term	*P*-value	Adjusted *P*-value	Odds ratio	Combined score	Genes
Melanoma	Ciclosporin MCF7 UP	3.24E-03	0.01	19,935	114,209.93	EDEM2
	Thapsigargin MCF7 UP	0.02	0.04	19,593	76,308.5	EDEM2
SCC	Vinzam BOSS	2.02E-05	0.01	493.04	5328.55	IL27; MAPK3
	Tetradioxin BOSS	3.69E-05	0.01	359.6	3670.45	IL27; MAPK3
	N-Formyl-Met-Leu-Phe BOSS	4.29E-05	0.01	332.58	3344.84	IL27; MAPK3
	Levamisole BOSS	4.50E-05	0.01	324.46	3247.67	IL27; MAPK3
	Platelet activating factor BOSS	5.85E-05	0.01	282.95	2757.65	IL27; MAPK3
	Zoledronic acid BOSS	6.34E-05	0.01	271.37	2622.95	IL27; MAPK3
	Telmisartan BOSS	9.71E-05	0.01	217.86	2013.05	IL27; MAPK3
	Ionomycin BOSS	1.00E-04	0.01	214.33	1973.7	IL27; MAPK3

SCC, squamous cell carcinoma; P-value, statistical significance value; Adjusted P-value, false discovery rate (FDR). BOSS, and UP, denote the specific experimental platform suffixes and annotation tags directly retrieved from the drug-gene connectivity database records used in this study. The Combined Score and Odds Ratio reflect the enrichment potency and association strength of the respective chemical compounds against the identified therapeutic target genes.

PheWAS revealed no significant associations with other traits, suggesting low side effect risk for BCC and SCC treatments (Supplementary Figures S46–S55).

### Single-cell-type analysis

Single-cell RNA-seq data from GEO were analyzed for cell type-specific enrichment of target genes.

**FIGURE 8:**
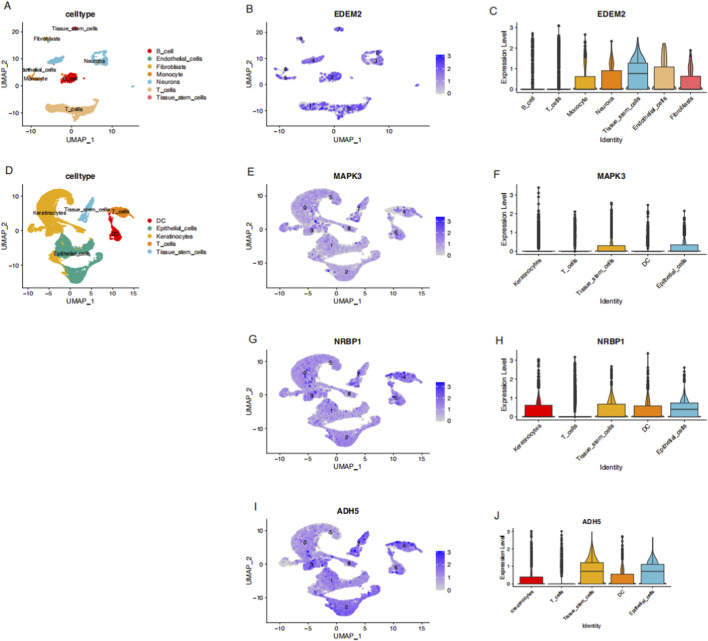
**(A,D)** The distinct cell types of SCC and melanoma single-cell analysis results. **(B,E,G,I)** The single-cell expression of these four genes in every cluster. **(C,F,H,J)** The expression level of four target genes (*EDEM2, MAPK3, NRBP1*, and *ADH5*) in cell types.

In SCC, 9 clusters yielded 5 cell types: epithelial cells, keratinocytes, tissue stem cells, T cells, and DCs (Figure 8D). In melanoma, 12 clusters produced 7 cell types: B cells, endothelial cells, fibroblasts, monocytes, neurons, T cells, and tissue stem cells (Figure 8A). *EDEM1*, *MAPK3*, *NRBP1*, and *ADH5* were expressed across all clusters; *IL27* and *ANKK1* were undetected (Figures 8B,E,G,I). Single-cell expression of these four genes is shown in Figures 8C,F,H,J.

## Discussion

This study integrated MR, FUMA GWAS, and SMR to examine modifiable risk factors for skin cancers (melanoma, SCC, and BCC) and identify potential therapeutic targets. By incorporating multi-omic data, we aimed to better understand the biological mechanisms underlying these targets. Drug prediction, PheWAS, and single-cell analysis were also conducted to guide the development of more effective therapies. Our integrated GWAS analysis linked AK to melanoma risk and showed that alcohol consumption was negatively associated with SCC. EDEM2 was identified as a potential drug target in melanoma, and five targets (ADH5, ANKK1, MAPK3, IL27, NRBP1) in SCC. Two compounds were predicted for melanoma treatment and over ten for SCC. Cellular analyses revealed the specific cell types targeted by these drugs in both cancers.

A more relaxed SNP selection threshold (*P* < 1 × 10^−5^) was used for actinic keratosis and HIV infection due to the limited availability of genome-wide significant variants. Although this strategy has been commonly used in MR studies involving traits with weak genetic architectures, it may increase the risk of weak instrument bias and should therefore be interpreted with caution.

The GWAS summary statistics used for Mendelian randomization analyses were obtained from publicly available studies consisting predominantly of individuals of European ancestry. For downstream biological interpretation, transcriptomic and single-cell RNA sequencing datasets were collected from independent cohorts. Because these datasets differed in population background, sample characteristics, tissue sources, and sequencing platforms, the single-cell analyses were primarily used to characterize cell-type-specific expression patterns and provide functional support for the identified candidate genes rather than to establish causality. Potential heterogeneity arising from cross-dataset integration was considered when interpreting the results.

Melanoma incidence has consistently increased, outpacing many cancers (Long et al., 2023). Prevention strategies focus on identifying individuals with phenotypic risk factors and reducing environmental exposures (Dzwierzynski, 2021). Consistently, recent bioinformatics and machine learning frameworks have underscored the necessity of integrating multi-omics data to uncover shared molecular properties, radiation or UV response pathways, and key hub biomarkers across both melanoma and non-melanoma skin cancers (Jannat et al., 2025). We identified a causal association between AK and melanoma risk (*OR* = 1.24, 95% *CI* 1.07–1.43, *P* < 0.01). This finding aligns with Bellinato et al. (2022), who reported that AK was associated with increased melanoma risk (Bellinato et al., 2022). A cohort study further observed that patients diagnosed with AK had a higher risk of developing melanoma within the decade following diagnosis (Guorgis et al., 2020). Elizabeth et al. also suggested that the presence of AK may indicate possible coexisting melanoma, warranting heightened clinical suspicion (Uhlenhake et al., 2010). Together, these studies provide strong evidence linking AK to melanoma risk.

Our investigation into AK as a risk factor for melanoma identified EDEM2 as a shared therapeutic target for both conditions. *EDEM2* was associated with melanoma risk, and its expression was negatively regulated by three methylation sites (cg20526800, cg04149172, cg05389922). EDEM2, a member of the EDEM family, possesses mannosidase activity and plays a critical role in glycoprotein degradation by performing the first rate-limiting mannose trimming step upstream of EDEM1 and EDEM3 (Ninagawa et al., 2014). It also serves as a key regulator of endoplasmic reticulum-associated glycoprotein degradation (ERAD), removing misfolded glycoproteins from the calnexin chaperone system (Bottos et al., 2016). Beyond its role in protein quality control, EDEM2 may influence melanoma through the degradation and trafficking of integrin alpha-1 and protocadherin 2 (Munteanu et al., 2021; Chirioiu et al., 2021). Based on these findings, we hypothesize that methylation-induced suppression of *EDEM2* disrupts ERAD and integrin trafficking in AK and melanoma cells, contributing to therapeutic effects. Drug prediction and single-cell analysis further suggested that ciclosporin and thapsigargin may target EDEM2-expressing endothelial cells, fibroblasts, monocytes, neurons, and tissue stem cells in melanoma.

We investigated two types of non-melanoma skin cancer: SCC and BCC. In contrast to our findings, Yu Sawada et al. reported that alcohol intake was positively associated with SCC risk (Sawada and Nakamura, 2021), whereas our analysis showed a negative association between drinks per week and SCC risk. A meta-analysis previously linked light drinking to esophageal SCC risk (Li et al., 2023b), supporting the plausibility of alcohol-related effects on squamous cell carcinogenesis. However, most current evidence comes from observational studies, which are highly susceptible to lifestyle confounders like smoking or outdoor UV exposure. Our MR analysis minimizes these environmental biases through genetic variants. Furthermore, low-to-moderate alcohol intake has been reported to paradoxically modulate systemic immunity and the gut-immune axis, partially via shifting microbial networks and generating acetate metabolites (Caslin et al., 2021), which may favorably alter the host anti-tumor immune response or tissue microenvironment. From a biological perspective, this genetic liability toward a protective tendency against SCC may be mediated by these systemic metabolic-immunological shifts. Peripheral acetate generated from mild alcohol metabolism can act as an important signaling molecule that reshapes host circulatory immunity, which could theoretically extend its anti-inflammatory or tumor-suppressive effects to the cutaneous microenvironment. Nonetheless, our primary purpose was not to advocate alcohol intake, but to leverage this genetic association to identify novel downstream druggable targets for SCC. Studies have shown that CDK10 is associated with malignant melanoma and BCC in unexposed skin (Bonilla et al., 2021), however, it failed in HEIDI test in SMR analysis and we excluded in subsequent analysis.

We identified five novel SCC targets (MAPK3, NRBP1, IL27, ADH5, ANKK1) and characterized their regulation at methylation, expression, and protein levels. Previous studies have identified MAPK3 as a potential drug target for esophageal squamous cell carcinoma (ESCC) and head and neck squamous cell carcinoma (HNSCC) (Sha et al., 2022; Zhang et al., 2022). In our study, *MAPK3* was regulated by three methylation sites (cg06295687, cg02767068, cg04060128) and negatively associated with SCC risk at both expression and protein levels, suggesting its potential role in SCC pathogenesis. *NRBP1* has been reported to be positively associated with ESCC risk, and its knockdown in an HNSCC cell line carrying an *NRBP1* mutation inhibited cell transformation and survival (Hwang et al., 2020; Chandrani et al., 2015). Similarly, in our analysis, *NRBP1* was negatively regulated by five methylation sites (cg01051318, cg23635560, cg08789022, cg01744354, cg06566627) and emerged as a potential target linked to SCC risk at expression and protein levels. *IL27* is overexpressed in oral squamous cell carcinoma (OSCC) (Wang et al., 2022), but exhibits antitumor effects in murine models: *IL-27* gene therapy suppressed NK-unsusceptible SCCVII tumors in a HNSCC model (Matsui et al., 2009). As a pleiotropic immune-regulatory cytokine, IL-27 acts as a double-edged sword in tumors, exhibiting both anti-tumor and pro-tumor activities depending on context (Maleki et al., 2025). A genetic variant in *ADH5* may increase susceptibility to ESCC in alcohol drinkers by down-regulating *ADH1A* and impairing alcohol metabolism (Cui et al., 2018). In our study, *ADH5* was intricately regulated by three methylation sites (cg21548116, cg08178063, cg23949936, cg15395354) and positively associated with SCC risk in individuals with high alcohol consumption. Drug prediction and single-cell analysis further revealed that compounds targeting IL27 and MAPK3 may act on epithelial cells, keratinocytes, tissue stem cells, and DC cells in SCC. Our pQTL-based SMR analysis confirmed that genetically elevated SULT1A3 protein levels are significantly linked to an increased risk of SCC. SULT1A3 is a prenatal-predominant cytosolic sulfotransferase (SULT) that can catalyze the bioactivation of pro-carcinogens (Dubaisi et al., 2019). We hypothesize that its aberrant upregulation in adult tissues drives SCC by inappropriately bioactivating environmental pro-carcinogens.

Our study has several advantages. First, using genetic variants in MR mitigates confounding and reverse causation. Second, integrating multiple approaches (meta-analysis, MR, SMR, and single-cell sequencing) enhanced statistical power. Third, to our knowledge, this is the first MR-based study to identify drug targets for skin cancers using the largest publicly available GWAS data on skin cancer risk. Overall, our multi-omic integration provides deeper insights into the biological mechanisms of these potential therapeutic targets. Nevertheless, several practical hurdles must be overcome for the clinical translation of these candidates. First, targeting ubiquitously expressed hub genes such as MAPK3 carries a potential risk of systemic on-target or off-target toxicities. Second, efficient tissue specific drug delivery to skin lesions remains a technical challenge that requires localized or topical formulation development. Finally, complex tumor heterogeneity and compensatory pathway activation may compromise the long-term efficacy of single target interventions.

Our study has limitations. First, to minimize confounding from population stratification, all genetic data were strictly restricted to populations of European ancestry; however, this homogeneous design inherently limits the direct generalizability of our findings to ethnically diverse populations. Second, our approach may lack sensitivity to detect small causal effects and assumes linear exposure-outcome relationships, which may not reflect high-dose clinical settings. Third, using blood eQTLs may not capture tissue-specific genetic regulation, limiting precision for treatment selection. Larger and more diverse GWAS datasets are needed for validation.

In conclusion, AK was positively associated with melanoma risk, while alcohol consumption was protective for SCC; no causal link was found for BCC. We identified EDEM2 as a melanoma target and five SCC targets (ADH5, ANKK1, MAPK3, IL27, NRBP1). These findings enhance skin cancer prevention and treatment strategies and provide a foundation for multi-omics guided targeted therapies. Further research and clinical trials on these genes are warranted.

## Data Availability

The original contributions presented in the study are included in the article/Supplementary Material, further inquiries can be directed to the corresponding author.
