# Supplementary material for: Modifiable risk factors and skin cancers: a multi-omics Mendelian randomization study from causal inference to drug target discovery
Source: Front Genet. 2026 Jul 7;17:1870725. doi: 10.3389/fgene.2026.1870725 (PMC13384438; doi:10.3389/fgene.2026.1870725)
Supplement: Supplementary file 1 [file DataSheet1.pdf]

Modifiable risk factors and skin cancers: a multi-omics Mendelian randomization study from causal inference to drug target discovery

Zhen Qin [1], Wuda Huoshen[2], Xueqing Li[2], Shiyu Li[2], Chen Sun[3], Sha Yi[4*]

[1]Department of Rheumatology and Immunology, The Affiliated Hospital, Southwest Medical University, Luzhou City, Sichuan Province, China

[2]School of Stomatology, Southwest Medical University, Luzhou, Sichuan, China

[3]Department of Periodontics and Oral Mucosal Diseases, The Affiliated Stomatology Hospital, Southwest Medical University, Luzhou, Sichuan, China

[4]Department of Dermatology, Chengdu Integrated TCM & Western Medicine Hospital, Chengdu, China.

[*]Correspondence: yisha890124@163.com

	BCC UKB	BCC FinnGen	Melanoma UKB	Melanoma FinnGen	SCC UKB	SCC FinnGen
Actinic keratosis	*P*=0.42	*P*=0.79	*P*=0.01	*P*=0.41	*P*=0.54	*P*=0.38
Age of Smoking Initiation	*P*=0.37	*P*=0.48	*P*=0.66	*P*=0.06	*P*=0.30	*P*=0.49
Cigarettes Per Day	*P*=0.58	*P*=0.97	*P*=0.84	*P*=0.77	*P*=3.09E-09	*P*=0.84
Drinks Per Week	*P*=0.41	*P*=0.68	*P*=0.77	*P*=0.22	*P*=0.02	*P*=0.53
HIV	*P*=0.31	*P*=0.94	*P*=0.54	*P*=0.38	*P*=0.69	*P*=0.11
Lifetime Smoking	*P*=0.85	*P*=0.23	*P*=0.57	*P*=0.01	*P*=4.58E-31	*P*=0.67
Nonionizing Radiation	*P*=0.55	*P*=0.69	*P*=0.11	*P*=0.51	P=0.17	*P*=0.21
Smoking Cessation	*P*=0.33	*P*=0.77	*P*=0.41	*P*=0.15	P=0.06	*P*=0.84
Smoking Initiation	*P*=0.85	*P*=0.78	*P*=0.81	*P*=0.87	*P*=1.24E-08	*P*=0.22
Sunburn	*P*=2.84E-36	*P*=2.49E-06	*P*=3.6E-09	*P*=0.01	*P*=1.02E-22	*P*=4.38E-06
Vitamin D	*P*=0.01	*P*=0.73	*P*=0.31	*P*=0.69	*P*=1.77E-04	*P*=0.35

Risk Factors
No Significance
Protective Factors

Figure S1. The IVW results of the discovery and replication cohorts before eliminating some results with pleiotropy from our analysis. RFs, the odds ratio (OR) > 1 and $P < 0.05$; No Significance, $P > 0.05$; Protective Factors, the OR < 1 and $P < 0.05$.

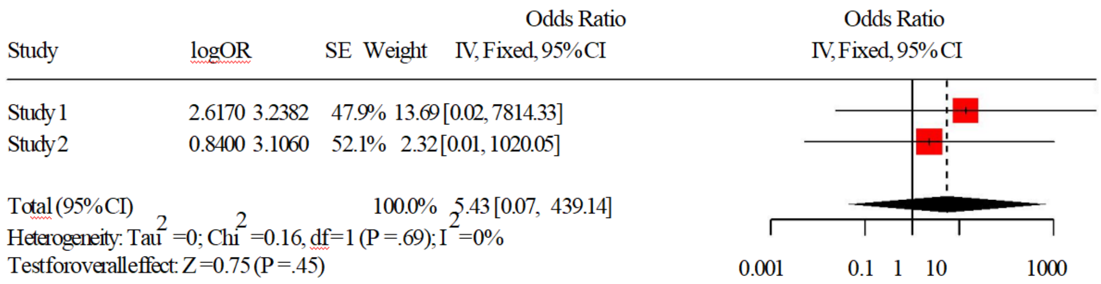

Figure S2. The meta-analysis result of actinic keratosis on BCC by using two data source. Study 1, UK Biobank data; Study 2,FinnGen data.

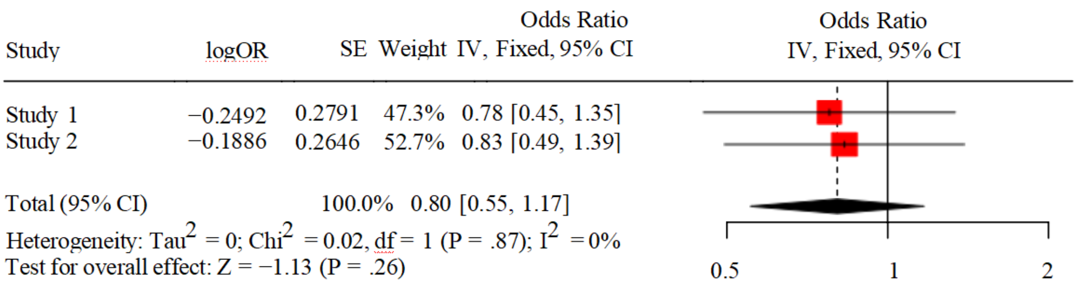

Figure S3. The meta-analysis result of age of initiation on BCC by using two data source. Study 1, UK Biobank data; Study 2,FinnGen data.

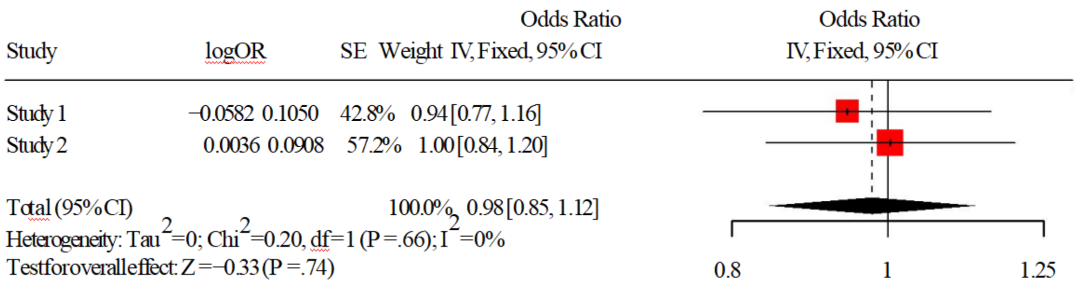

Figure S4. The meta-analysis result of cigarettes per day on BCC by using two data source. Study 1, UK Biobank data; Study 2,FinnGen data.

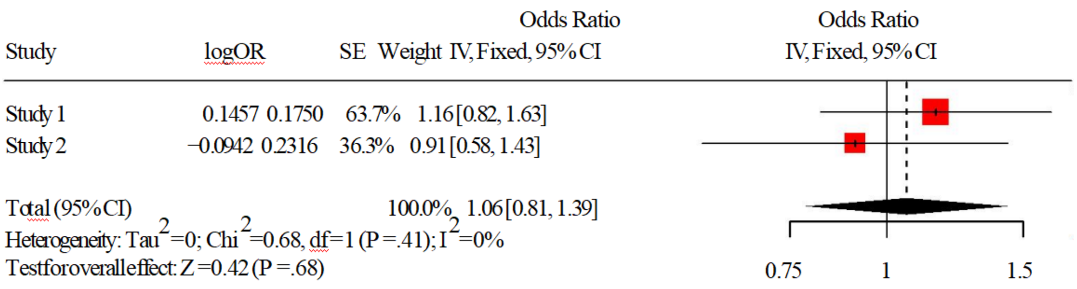

Figure S5. The meta-analysis result of drinks per week on BCC by using two data source. Study 1, UK

Biobank data; Study 2,FinnGen data.

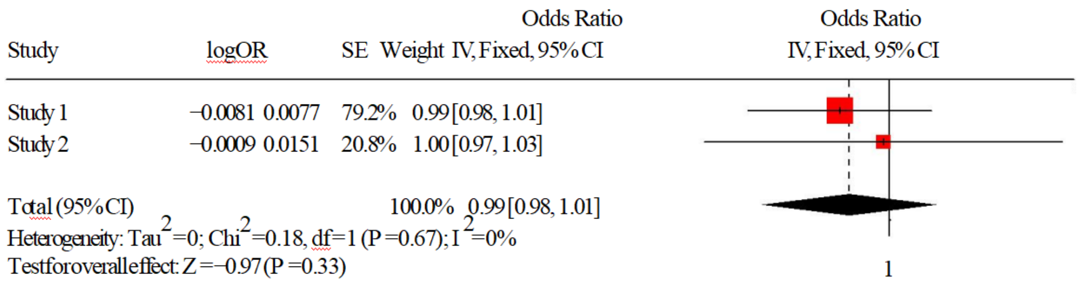

Figure S6. The meta-analysis result of HIV on BCC by using two data source. Study 1, UK Biobank data; Study 2,FinnGen data.

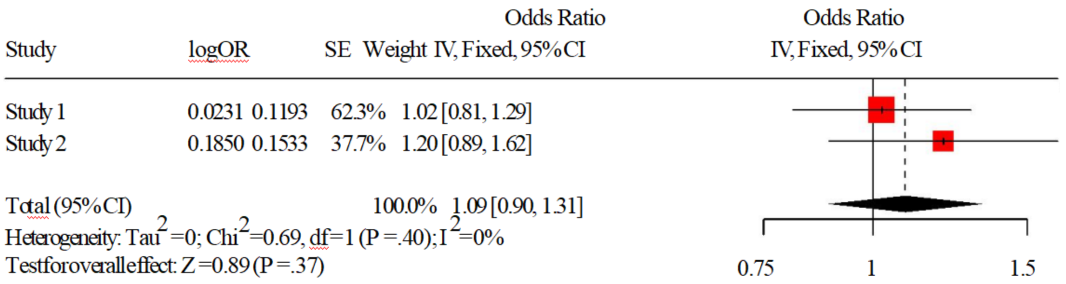

Figure S7. The meta-analysis result of lifetime smoking on BCC by using two data source. Study 1, UK Biobank data; Study 2,FinnGen data.

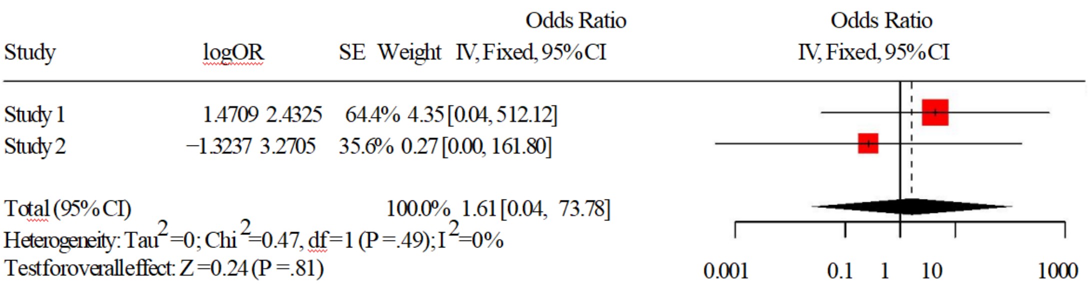

Figure S8. The meta-analysis result of nonionizing radiation on BCC by using two data source. Study 1, UK Biobank data; Study 2,FinnGen data.

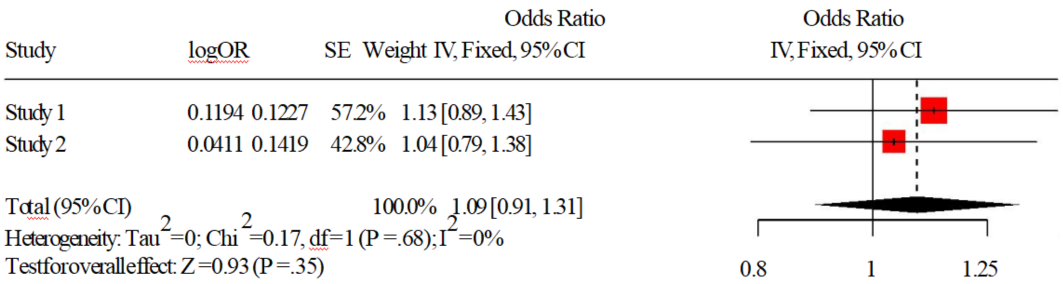

Figure S9. The meta-analysis result of smoking cessation on BCC by using two data source. Study 1, UK

Biobank data; Study 2,FinnGen data.

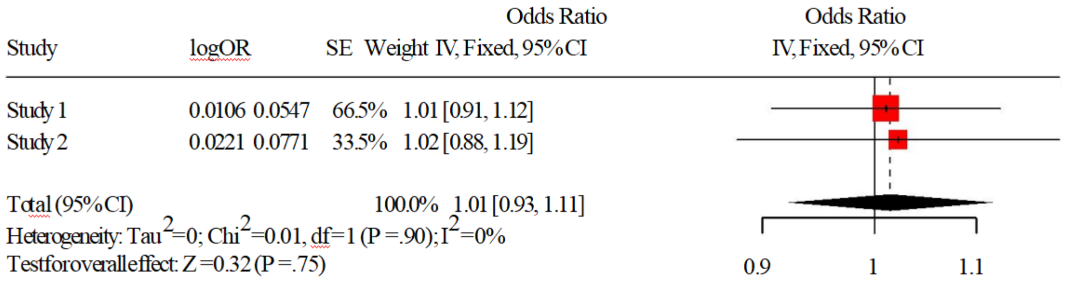

Figure S10. The meta-analysis result of smoking initiation on BCC by using two data source. Study 1, UK Biobank data; Study 2,FinnGen data.

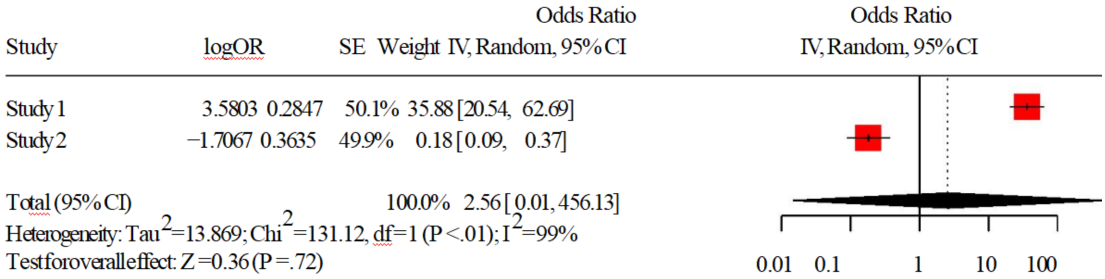

Figure S11. The meta-analysis result of sunburn on BCC by using two data source. Study 1, UK Biobank data; Study 2,FinnGen data.

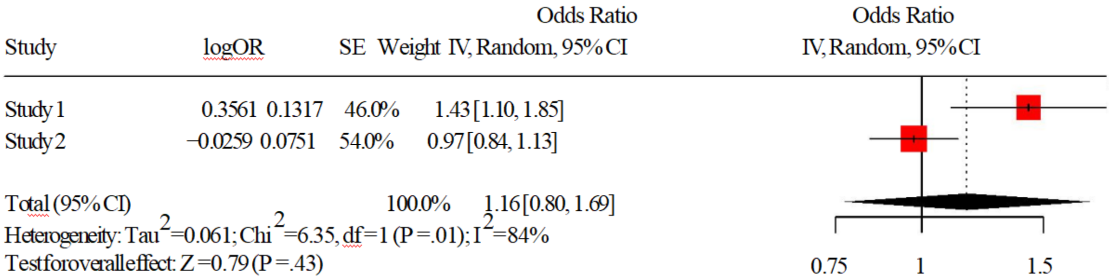

Figure S12. The meta-analysis result of Vitamin D on BCC by using two data source. Study 1, UK Biobank data; Study 2,FinnGen data.

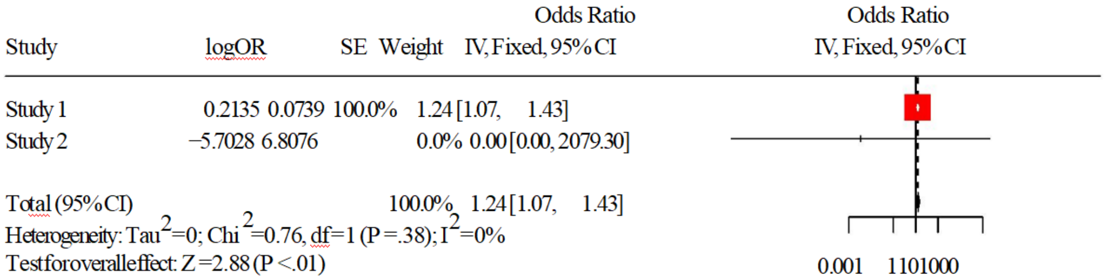

Figure S13. The meta-analysis result of actinic keratosis on melanoma by using two data source. Study

1, UK Biobank data; Study 2,FinnGen data.

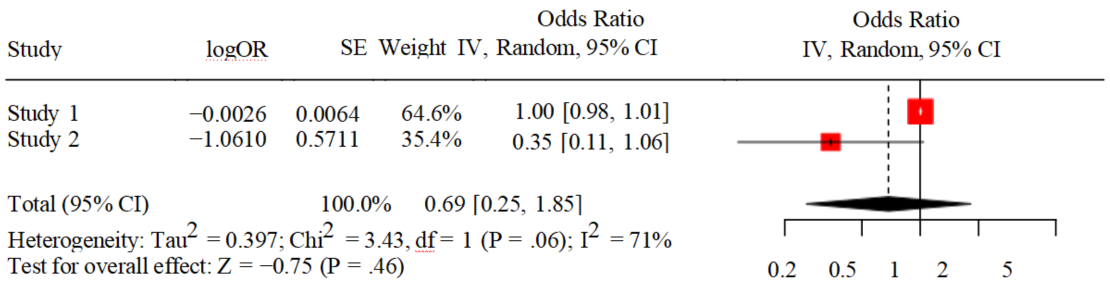

Figure S14. The meta-analysis result of age of initiation on melanoma by using two data source. Study 1, UK Biobank data; Study 2,FinnGen data.

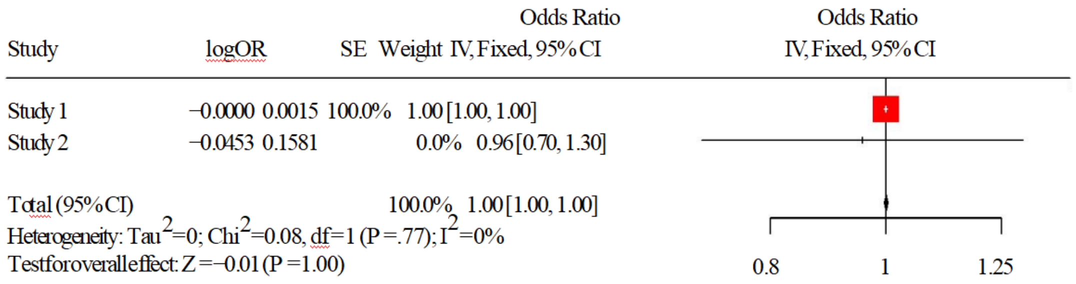

Figure S15. The meta-analysis result of cigarettes per day on melanoma by using two data source. Study 1, UK Biobank data; Study 2,FinnGen data.

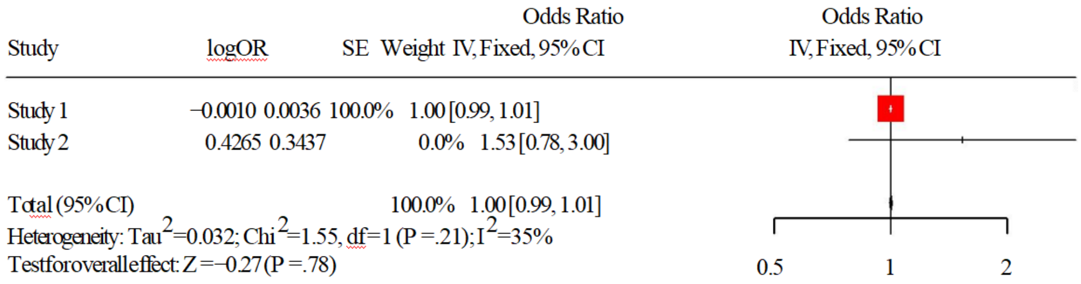

Figure S16. The meta-analysis result of drinks per week on melanoma by using two data source. Study 1, UK Biobank data; Study 2,FinnGen data.

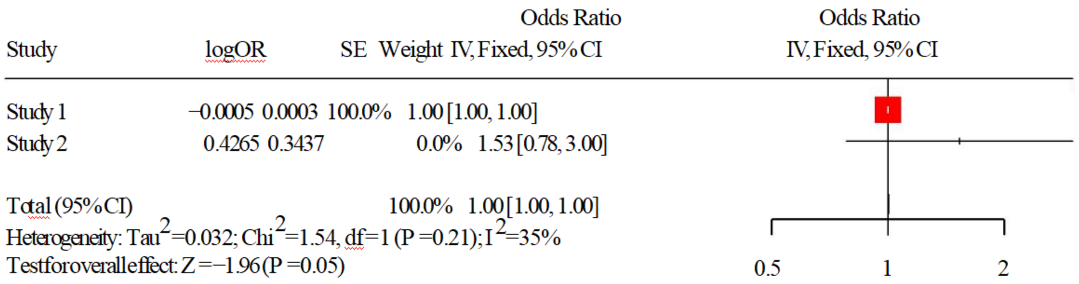

Figure S17. The meta-analysis result of HIV on melanoma by using two data source. Study 1, UK

Biobank data; Study 2,FinnGen data.

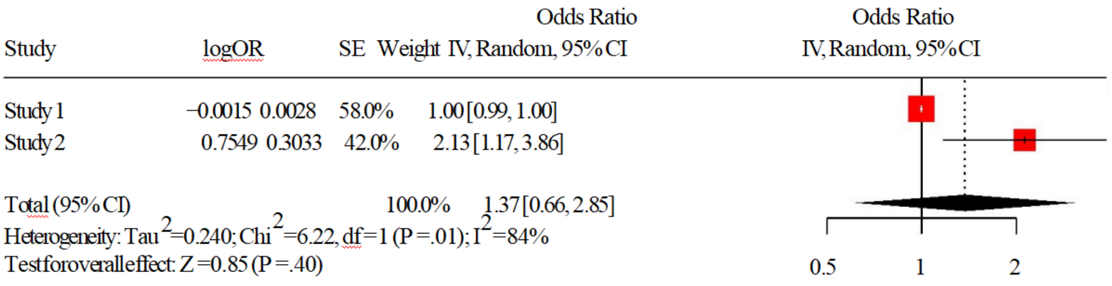

Figure S18. The meta-analysis result of lifetime smoking on melanoma by using two data source. Study 1, UK Biobank data; Study 2,FinnGen data.

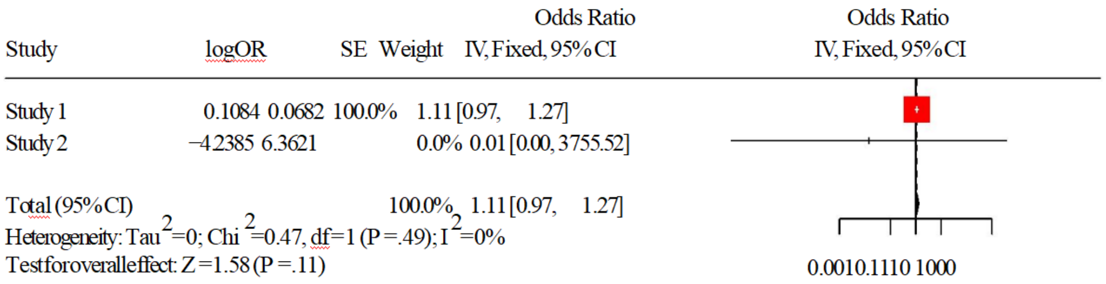

Figure S19. The meta-analysis result of nonionizing radiation on melanoma by using two data source. Study 1, UK Biobank data; Study 2,FinnGen data.

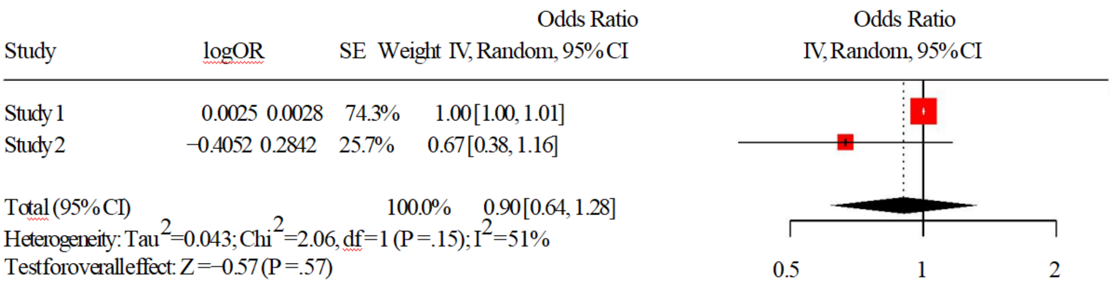

Figure S20. The meta-analysis result of smoking cessation on melanoma by using two data source. Study 1, UK Biobank data; Study 2,FinnGen data.

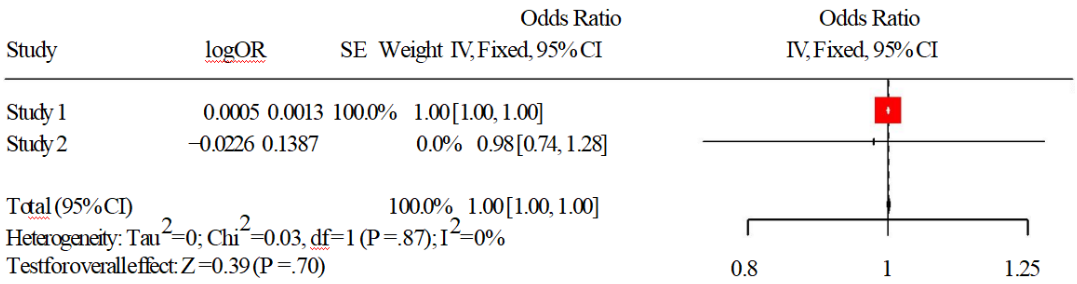

Figure S21. The meta-analysis result of smoking initiation on melanoma by using two data source. Study

1, UK Biobank data; Study 2,FinnGen data.

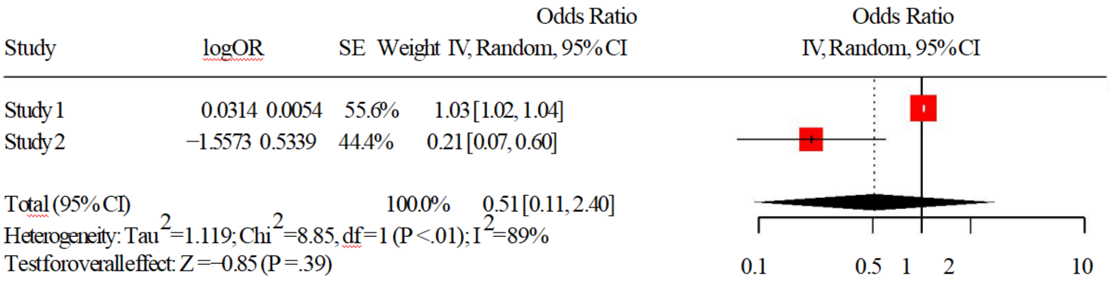

Figure S22. The meta-analysis result of sunburn on melanoma by using two data source. Study 1, UK Biobank data; Study 2,FinnGen data.

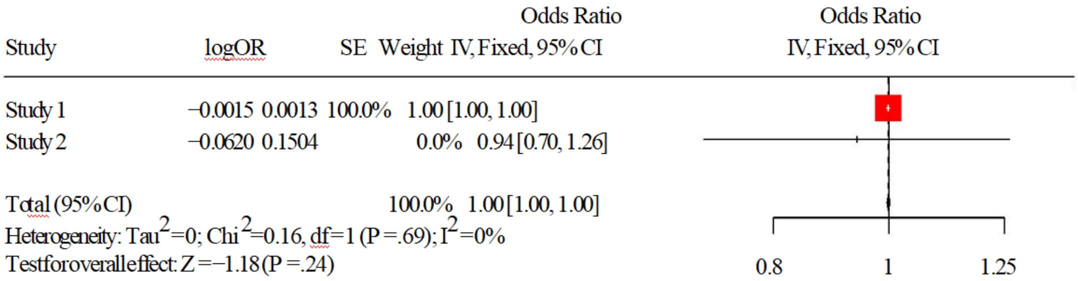

Figure S23. The meta-analysis result of Vitamin D on melanoma by using two data source. Study 1, UK Biobank data; Study 2,FinnGen data.

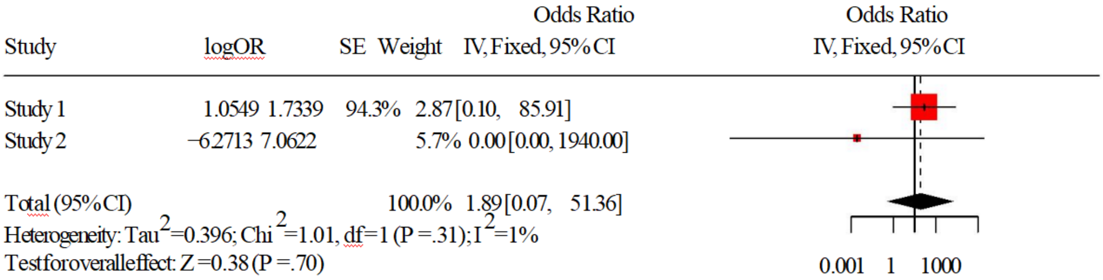

Figure S24. The meta-analysis result of actinic keratosis on SCC by using two data source. Study 1, UK Biobank data; Study 2,FinnGen data.

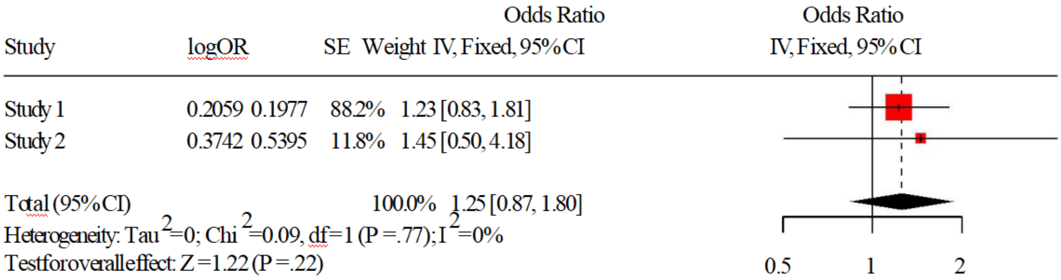

Figure S25. The meta-analysis result of age of initiation on SCC by using two data source. Study 1, UK

Biobank data; Study 2,FinnGen data.

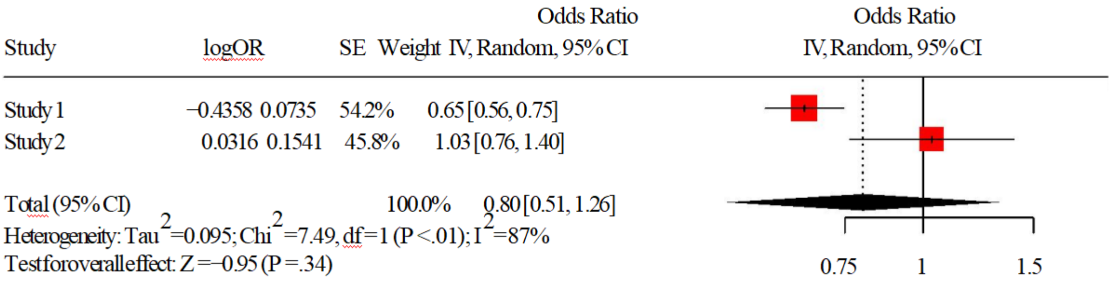

Figure S26. The meta-analysis result of cigarettes per day on SCC by using two data source. Study 1, UK Biobank data; Study 2,FinnGen data.

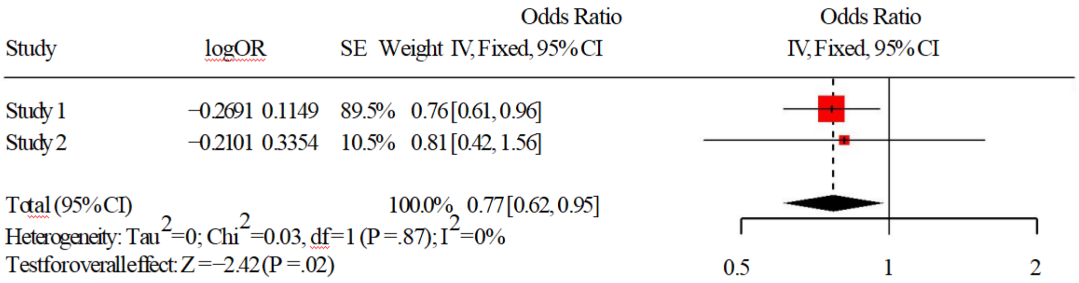

Figure S27. The meta-analysis result of drinks per week on SCC by using two data source. Study 1, UK Biobank data; Study 2,FinnGen data.

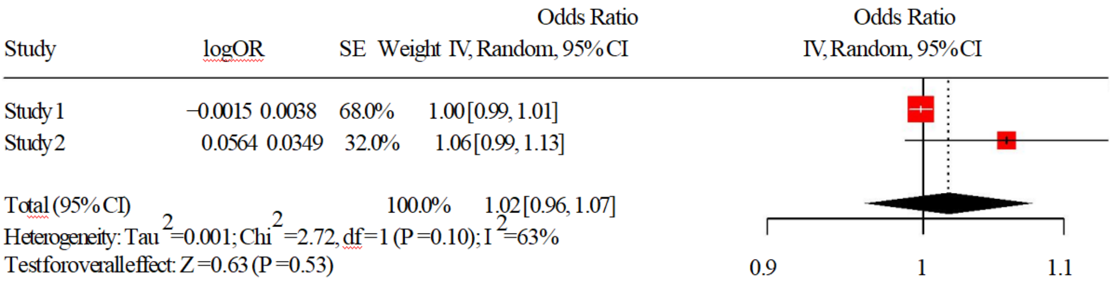

Figure S28. The meta-analysis result of HIV on SCC by using two data source. Study 1, UK Biobank data; Study 2,FinnGen data.

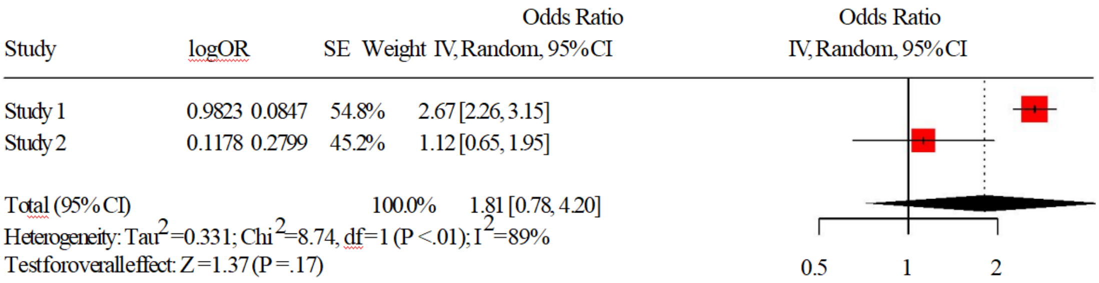

Figure S29. The meta-analysis result of lifetime smoking on SCC by using two data source. Study 1, UK

Biobank data; Study 2,FinnGen data.

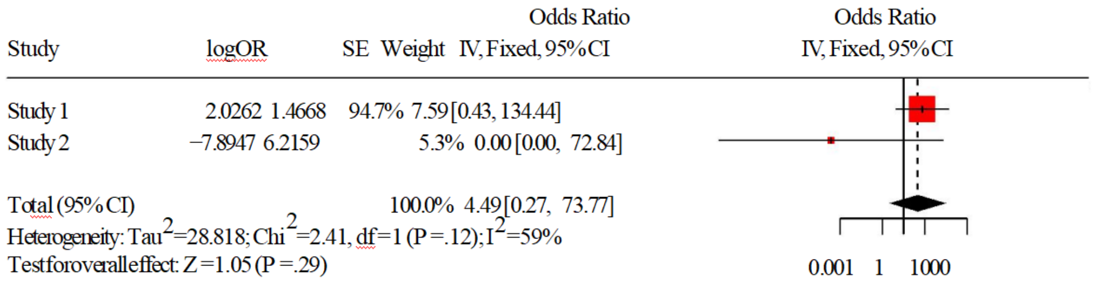

Figure S30. The meta-analysis result of nonionizing radiation on SCC by using two data source. Study 1, UK Biobank data; Study 2,FinnGen data.

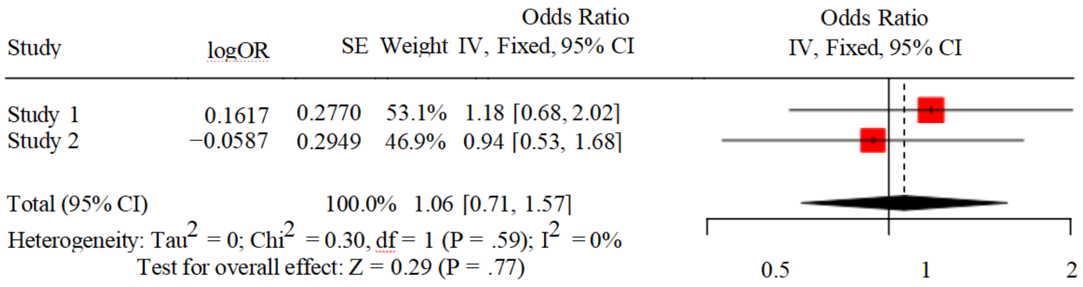

Figure S31. The meta-analysis result of smoking cessation on SCC by using two data source. Study 1, UK Biobank data; Study 2,FinnGen data.

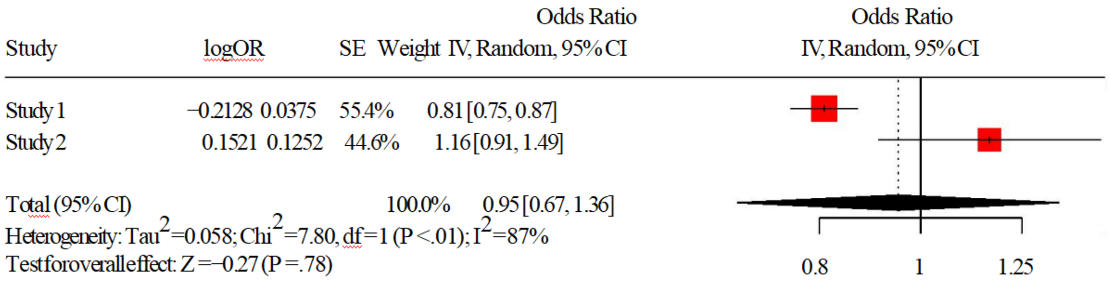

Figure S32. The meta-analysis result of smoking initiation on SCC by using two data source. Study 1, UK Biobank data; Study 2,FinnGen data.

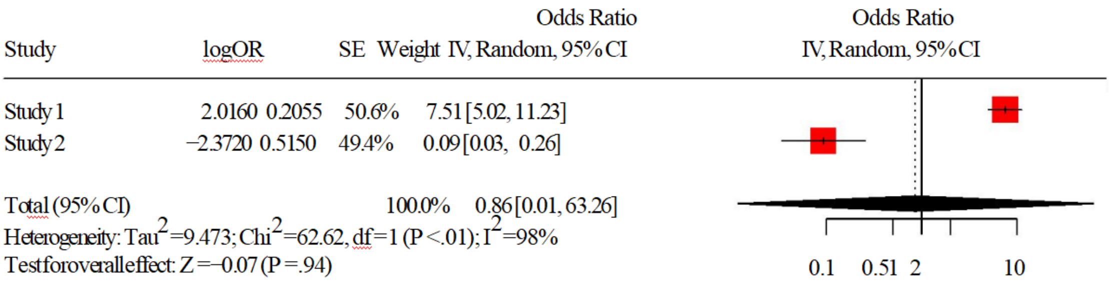

Figure S33. The meta-analysis result of sunburn on SCC by using two data source. Study 1, UK Biobank

data; Study 2,FinnGen data.

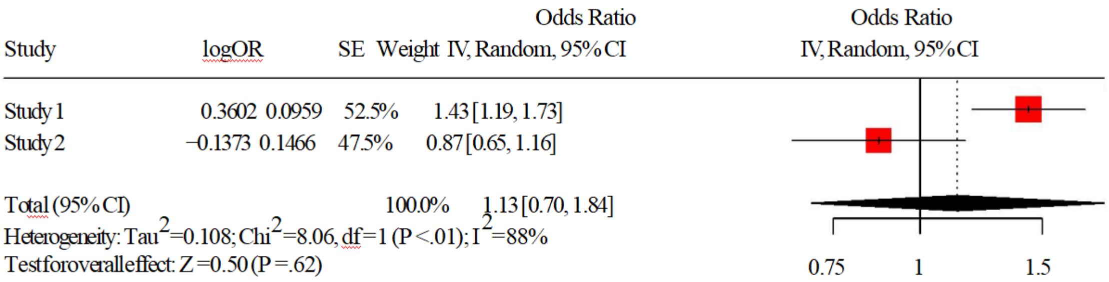

Figure S34. The meta-analysis result of Vitamin D on SCC by using two data source. Study 1, UK Biobank data; Study 2,FinnGen data.

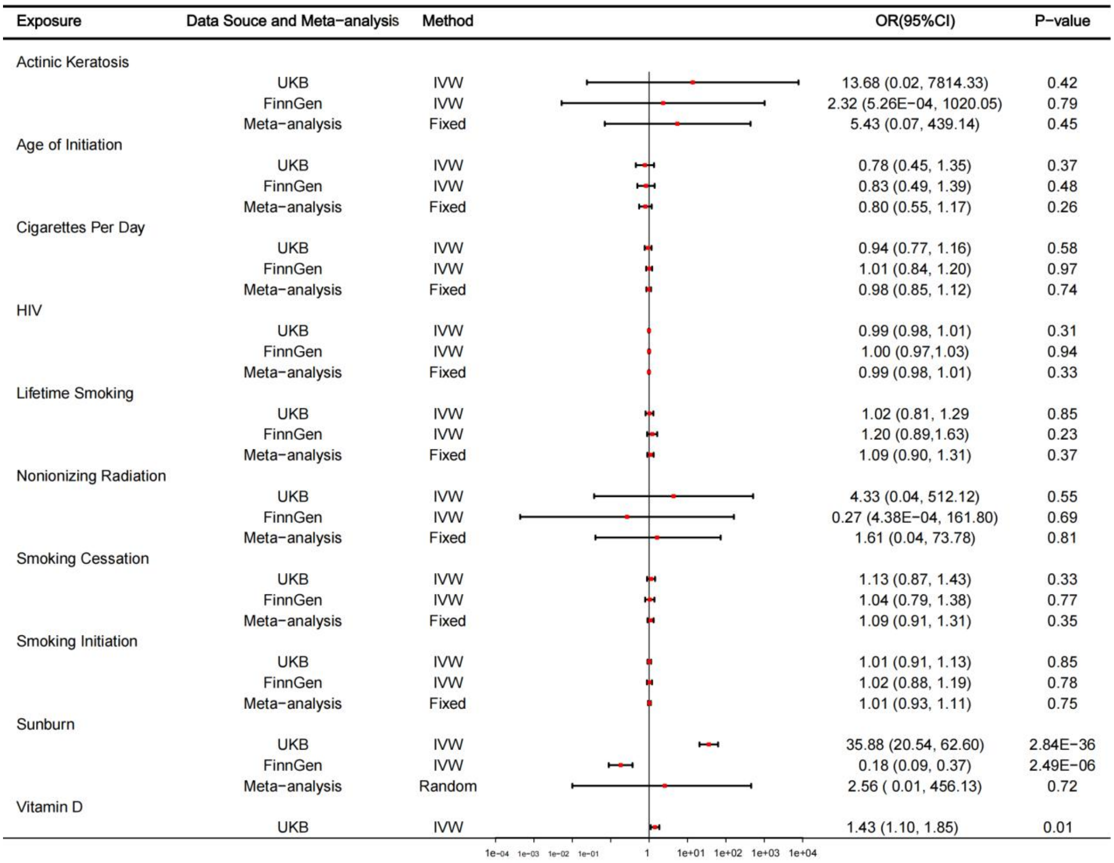

Figure S35. The final casual association between modifiable RFs and BCC after meta-analysis for the outcomes.

Exposure	Data Souce and Meta−analysis	Method	OR(95%CI)	P−value
Actinic Keratosis				
	UKB	IVW	2.87 (0.10, 85.91)	0.54
	FinnGen	IVW	0.01 (1.84E−09, 1940.00)	0.38
	Meta−analysis	Fixed	1.89 (0.07, 51.36)	0.70
Age of Initiation				
	UKB	IVW	1.23 (0.83, 1.81)	0.30
	FinnGen	IVW	1.45 (0.51, 4.19)	0.49
	Meta−analysis	Fixed	1.25 (0.87, 1.80)	0.22
Cigarettes Per Day				
	UKB	IVW	0.65 (0.56, 0.78)	3.09E−09
	FinnGen	IVW	1.03 (0.76, 1.40)	0.84
	Meta−analysis	Random	0.80 (0.51, 1.26)	0.34
Drinks Per Week				
	UKB	IVW	0.76 (0.61, 0.96)	0.02
	FinnGen	IVW	0.81 (0.42, 1.56)	0.53
	Meta−analysis	Fixed	0.77 (0.62, 0.95)	0.02
HIV				
	UKB	IVW	1.00 (0.99, 1.01)	0.69
	FinnGen	IVW	1.06 (0.99, 1.13)	0.11
	Meta−analysis	Random	1.02 (0.96, 1.13)	0.53
Lifetime Smoking				
	UKB	IVW	2.67 (2.26, 3.15)	4.58E−31
	FinnGen	IVW	1.13 (0.65, 1.95)	0.67
	Meta−analysis	Random	1.81 (0.78, 4.20)	0.17
Nonionizing Radiation				
	UKB	IVW	7.58 (0.43, 134.44)	0.17
	FinnGen	IVW	3.73E−04 (1.91E−09, 72.84)	0.20
	Meta−analysis	Fixed	4.49 (0.27, 73.77)	0.29
Smoking Cessation				
	UKB	IVW	1.18 (0.68, 2.02)	0.06
	FinnGen	IVW	0.94(0.53, 1.68)	0.84
	Meta−analysis	Fixed	1.06 (0.71, 1.57)	0.77
Smoking Initiation				
	UKB	IVW	0.81 (0.75, 0.87)	1.24E−08
	FinnGen	IVW	1.17 (0.91, 1.49)	0.22
	Meta−analysis	Random	0.95 (0.67, 1.36)	0.78
Sunburn				
	UKB	IVW	7.51 (5.02, 11.23)	1.02E−22
	FinnGen	IVW	0.09 (0.03, 0.26)	4.38E−06
	Meta−analysis	Random	0.86 (0.01, 63.26)	0.94
Vitamin D				
	UKB	IVW	1.43 (1.19, 1.73)	1.77E−04
	FinnGen	IVW	0.87 (0.65, 1.16)	0.35
	Meta−analysis	Random	1.13 (0.70, 1.84)	0.62

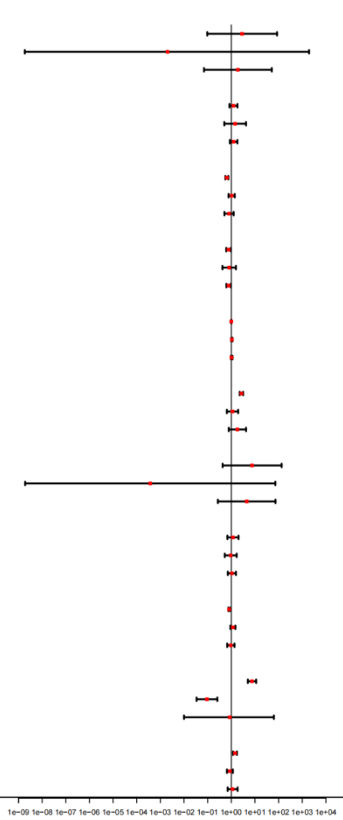

Figure S36. The final casual association between modifiable RFs and SCC after meta-analysis for the outcomes.

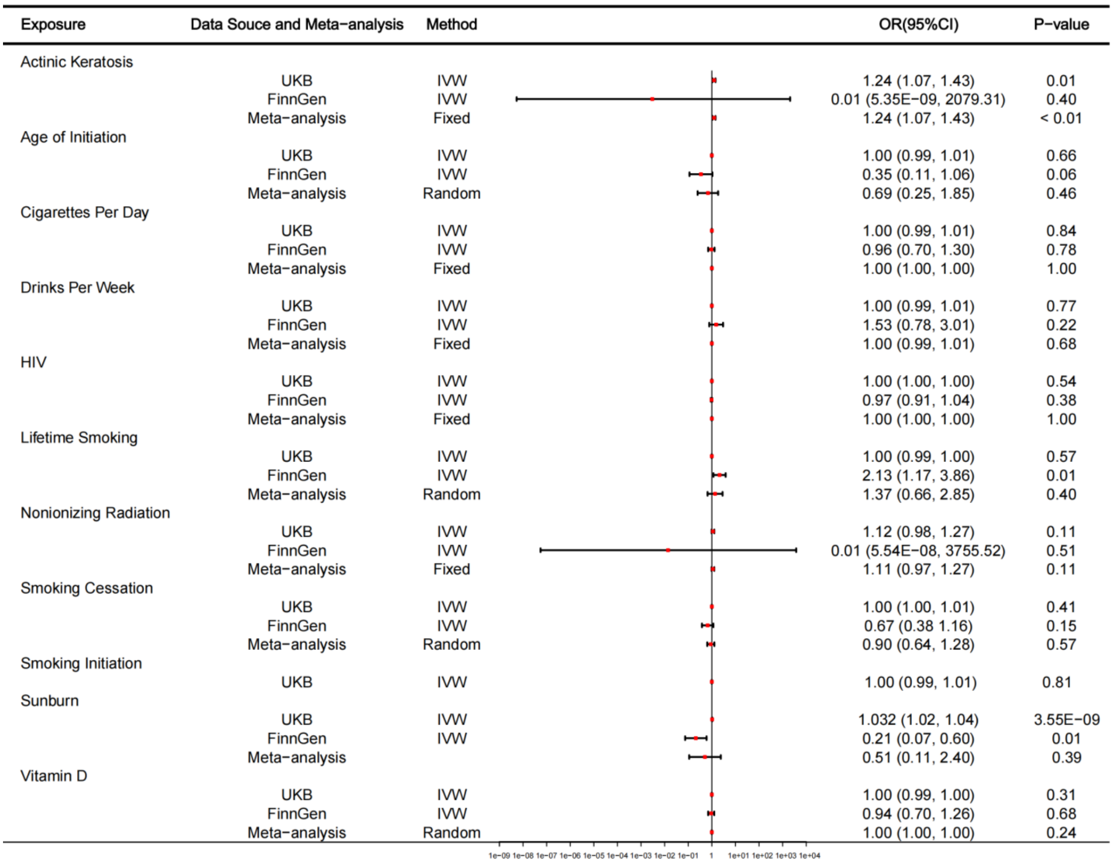

Exposure	Data Souce and Meta−analysis	Method	OR(95%CI)	P−value
Actinic Keratosis				
	UKB	IVW	1.24 (1.07, 1.43)	0.01
	FinnGen	IVW	0.01 (5.35E−09, 2079.31)	0.40
	Meta−analysis	Fixed	1.24 (1.07, 1.43)	< 0.01
Age of Initiation				
	UKB	IVW	1.00 (0.99, 1.01)	0.66
	FinnGen	IVW	0.35 (0.11, 1.06)	0.06
	Meta−analysis	Random	0.69 (0.25, 1.85)	0.46
Cigarettes Per Day				
	UKB	IVW	1.00 (0.99, 1.01)	0.84
	FinnGen	IVW	0.96 (0.70, 1.30)	0.78
	Meta−analysis	Fixed	1.00 (1.00, 1.00)	1.00
Drinks Per Week				
	UKB	IVW	1.00 (0.99, 1.01)	0.77
	FinnGen	IVW	1.53 (0.78, 3.01)	0.22
	Meta−analysis	Fixed	1.00 (0.99, 1.01)	0.68
HIV				
	UKB	IVW	1.00 (1.00, 1.00)	0.54
	FinnGen	IVW	0.97 (0.91, 1.04)	0.38
	Meta−analysis	Fixed	1.00 (1.00, 1.00)	1.00
Lifetime Smoking				
	UKB	IVW	1.00 (0.99, 1.00)	0.57
	FinnGen	IVW	2.13 (1.17, 3.86)	0.01
	Meta−analysis	Random	1.37 (0.66, 2.85)	0.40
Nonionizing Radiation				
	UKB	IVW	1.12 (0.98, 1.27)	0.11
	FinnGen	IVW	0.01 (5.54E−08, 3755.52)	0.51
	Meta−analysis	Fixed	1.11 (0.97, 1.27)	0.11
Smoking Cessation				
	UKB	IVW	1.00 (1.00, 1.01)	0.41
	FinnGen	IVW	0.67 (0.38 1.16)	0.15
	Meta−analysis	Random	0.90 (0.64, 1.28)	0.57
Smoking Initiation				
	UKB	IVW	1.00 (0.99, 1.01)	0.81
Sunburn				
	UKB	IVW	1.032 (1.02, 1.04)	3.55E−09
	FinnGen	IVW	0.21 (0.07, 0.60)	0.01
	Meta−analysis		0.51 (0.11, 2.40)	0.39
Vitamin D				
	UKB	IVW	1.00 (0.99, 1.00)	0.31
	FinnGen	IVW	0.94 (0.70, 1.26)	0.68
	Meta−analysis	Random	1.00 (1.00, 1.00)	0.24

Figure S37. The final casual association between modifiable RFs and melanoma after meta-analysis for the outcomes.

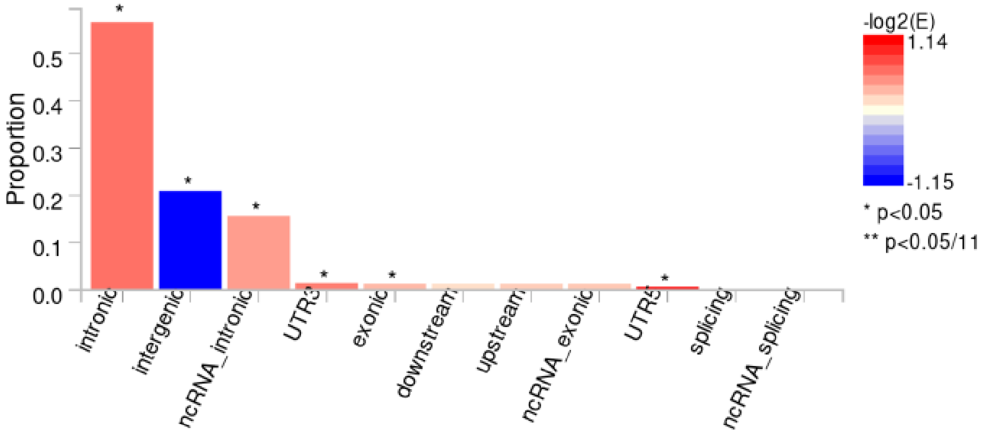

Figure S38. Functional consequences of SNPs on genes in drinks per week.

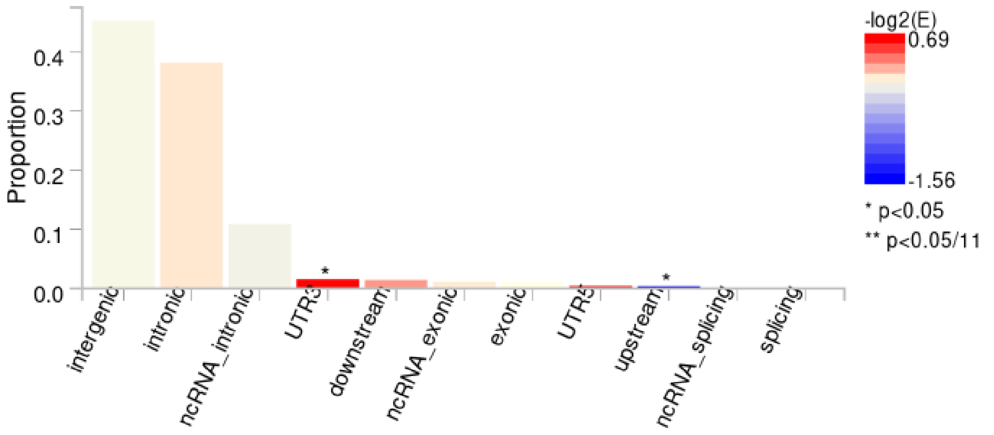

Figure S39. Functional consequences of SNPs on genes in actinic keratosis.

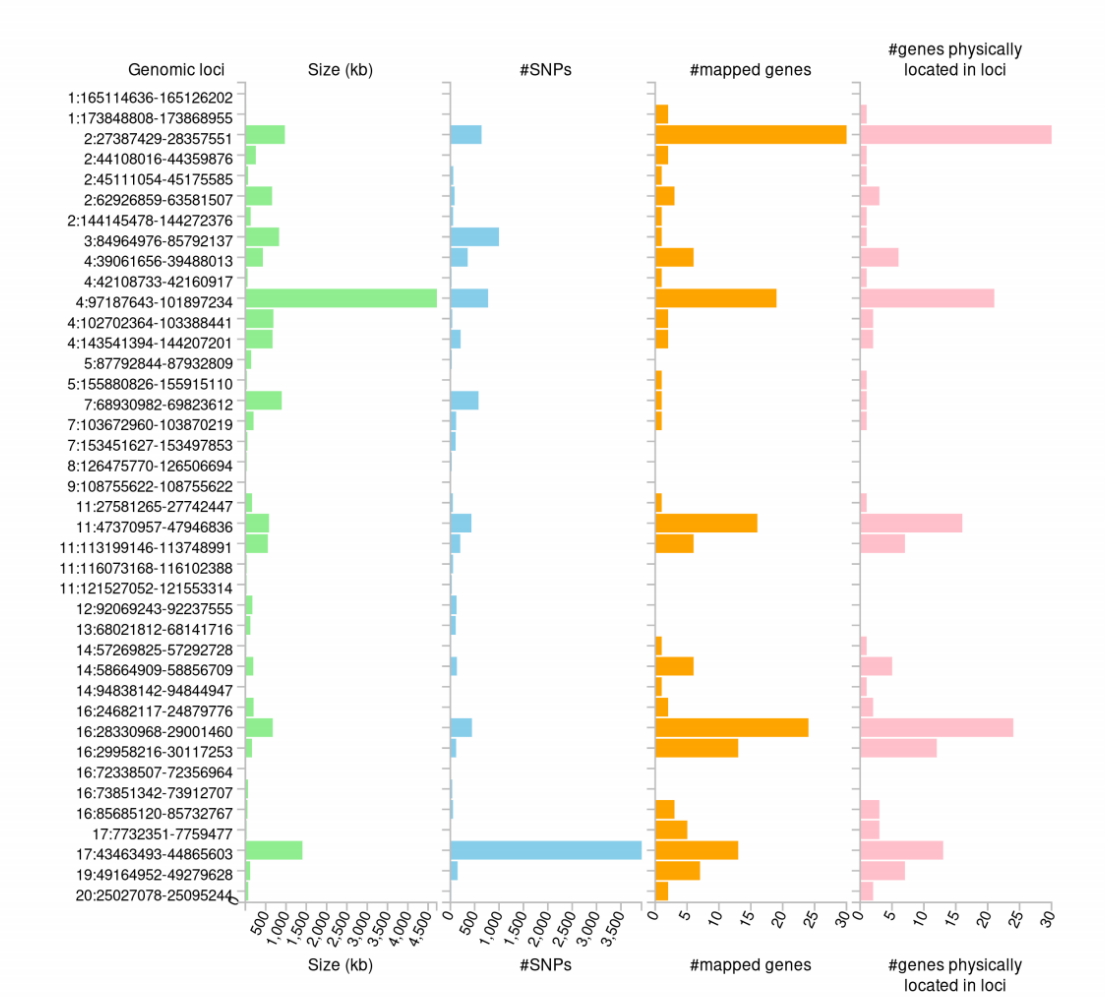

Figure S40. Summary per genomic risk locus in drinks per week.

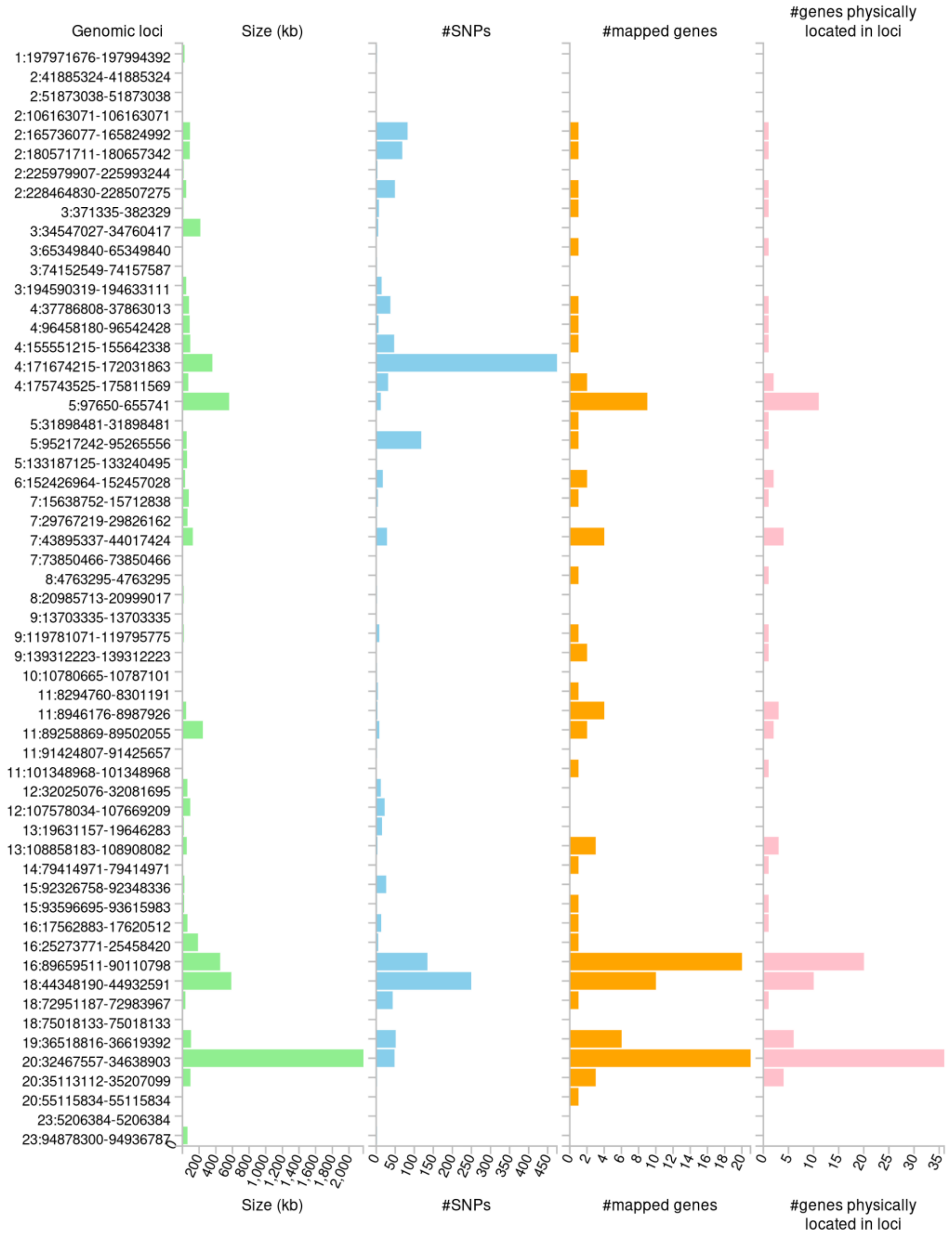

Figure S41. Summary per genomic risk locus in actinic keratosis.

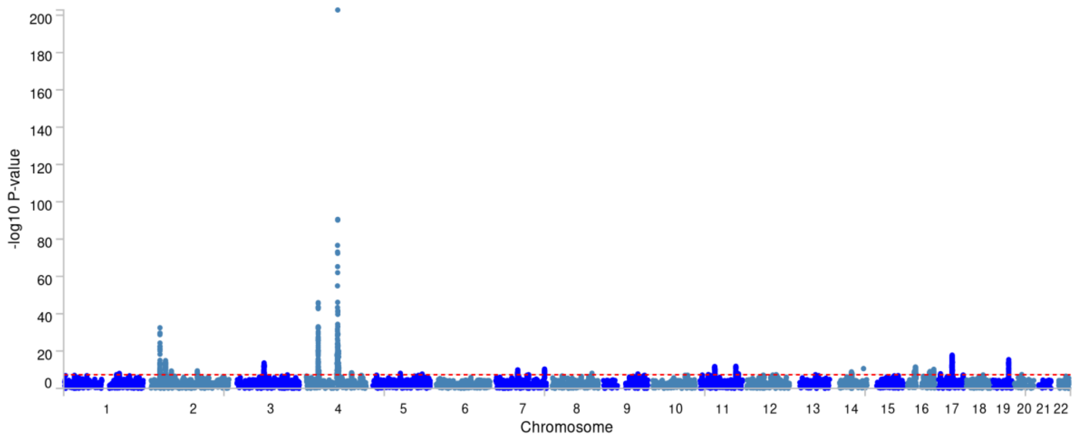

Figure S42.The manhattan plot of the gene-based test as computed by MAGMA based on input GWAS summary statistics of drinks per week.

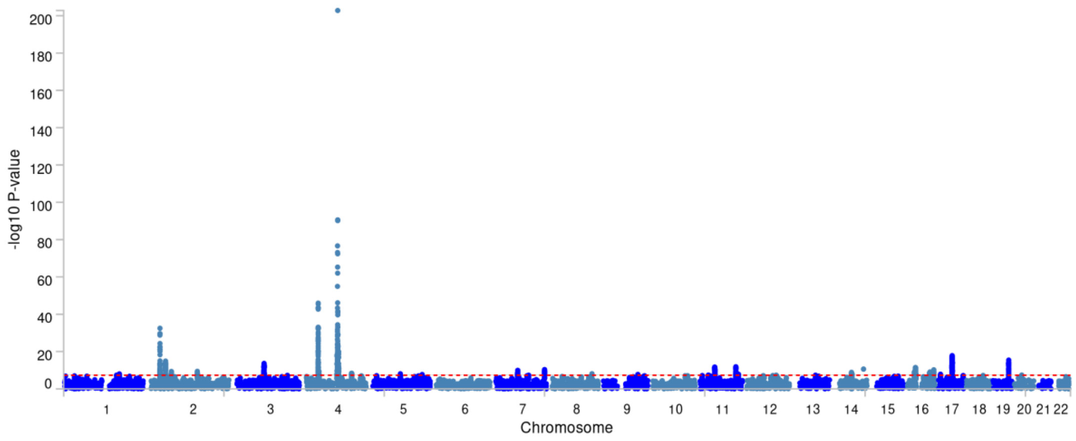

Figure S43.The manhattan plot of the gene-based test as computed by MAGMA based on input GWAS summary statistics of actinic keratosis.

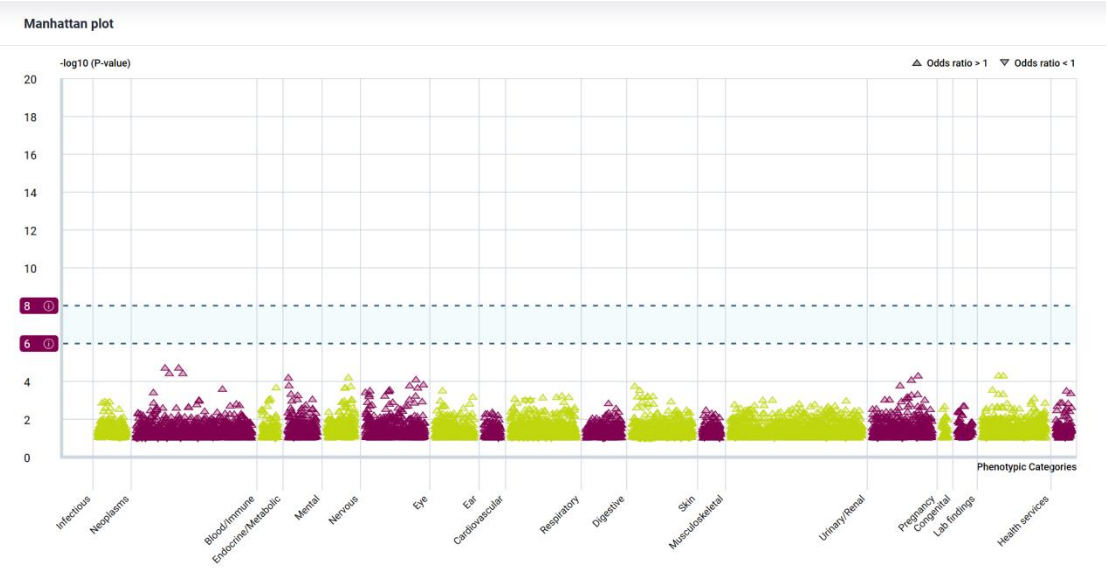

Figure S44. The manhattan plot of continuous traits PheWAS association with ADH5.

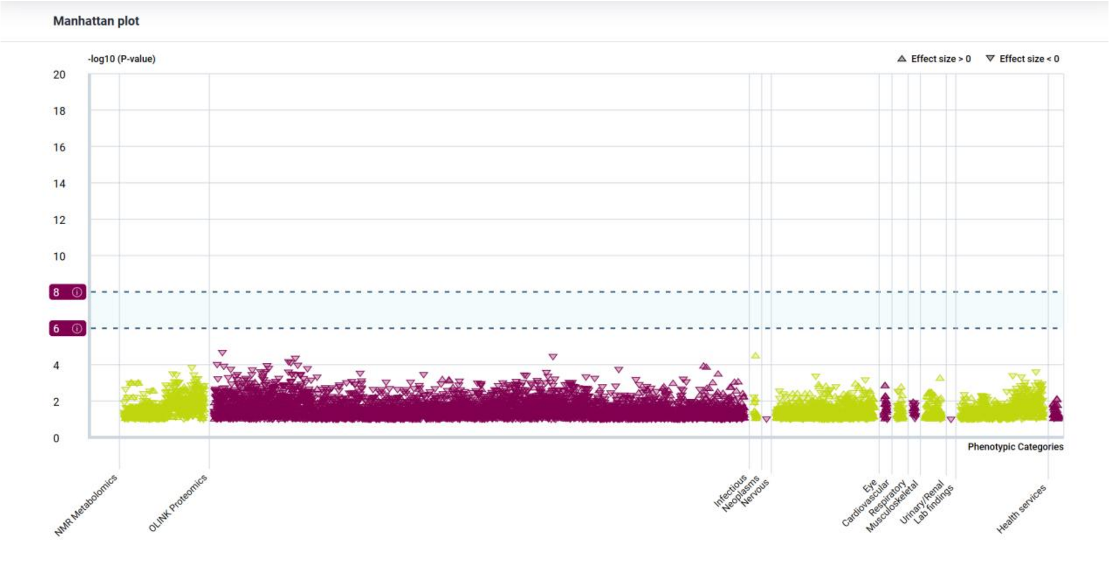

Figure S45. The manhattan plot of continuous traits PheWAS association with ADH5.

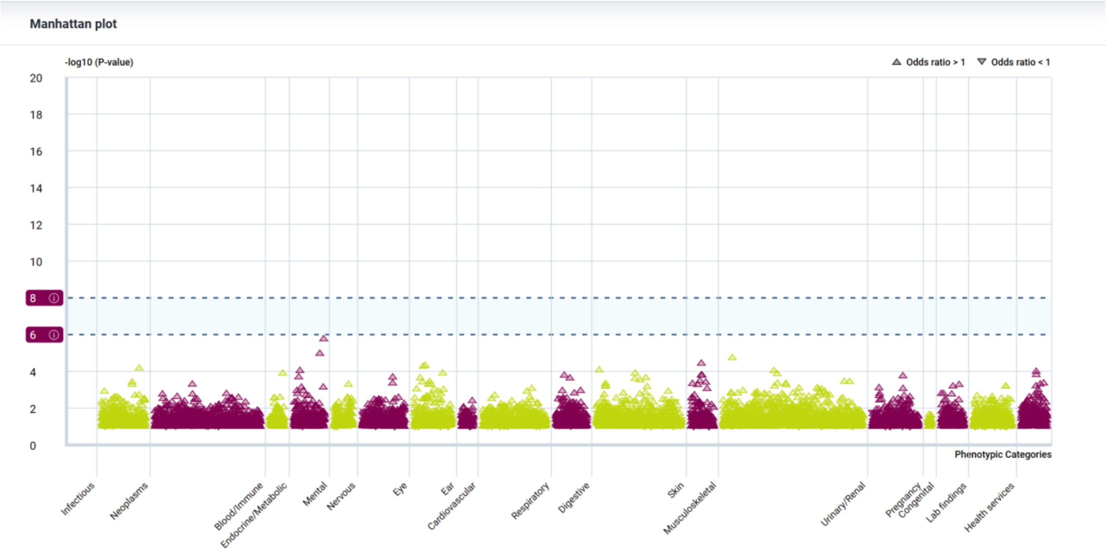

Figure S46. The manhattan plot of binary traits PheWAS association with ANKK1.

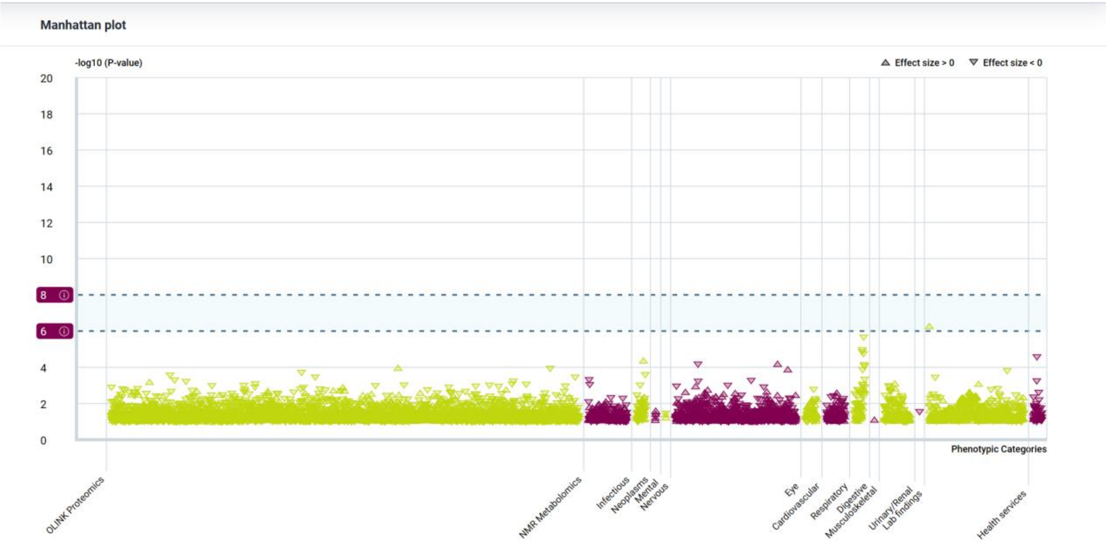

Figure S47. The manhattan plot of continuous traits PheWAS association with ANKK1.

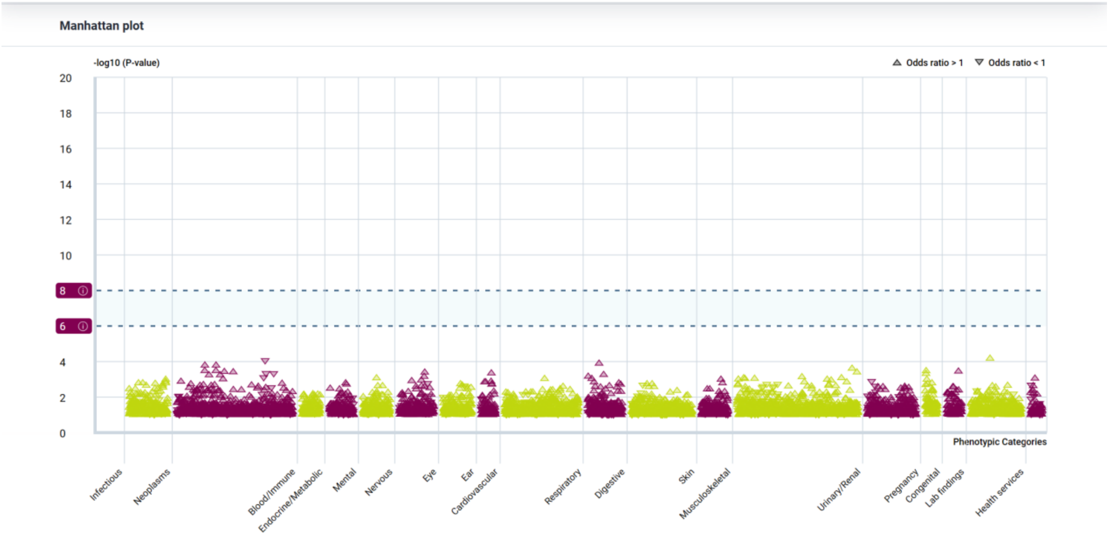

Figure S48. The manhattan plot of binary traits PheWAS association with EDEM2.

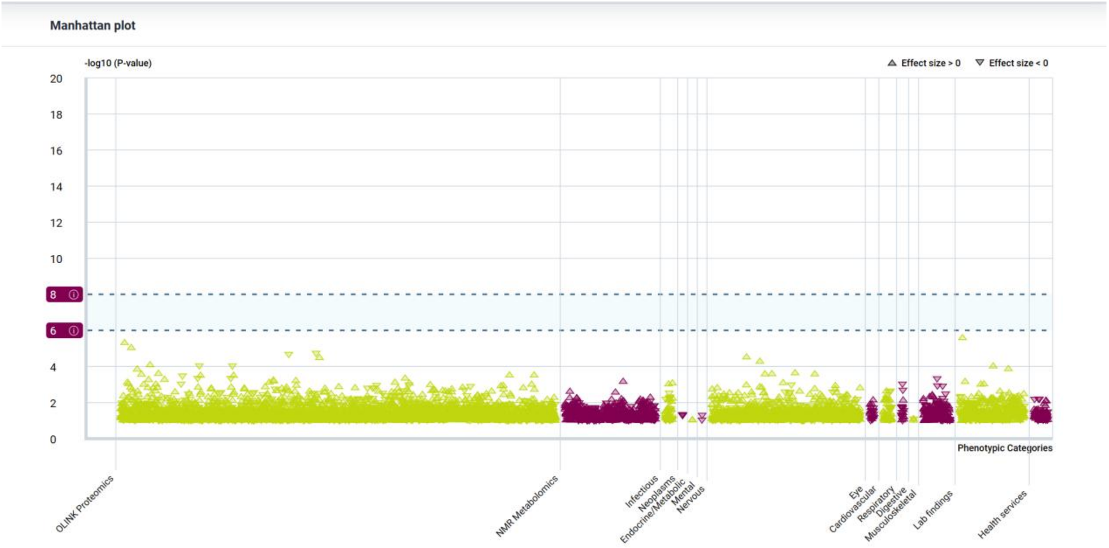

Figure S49. The manhattan plot of continuous traits PheWAS association with EDEM2.

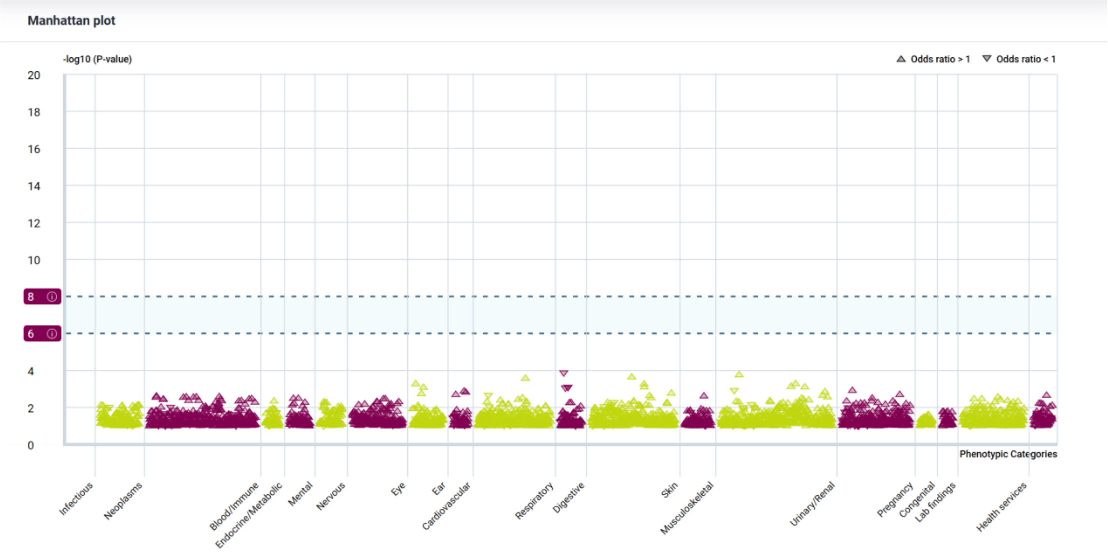

Figure S50. The manhattan plot of binary traits PheWAS association with IL27.

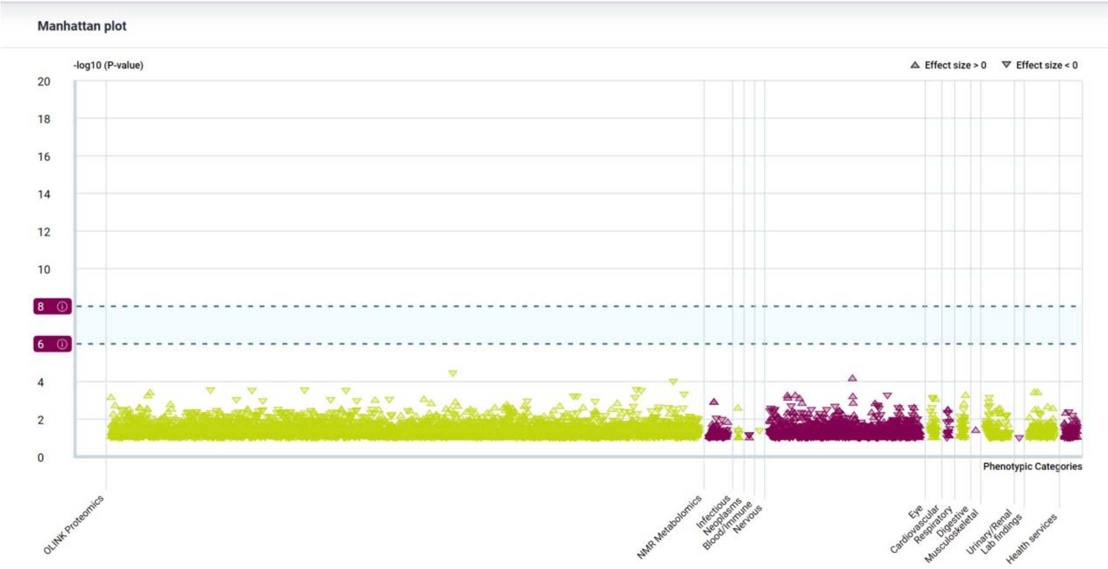

Figure S51. The manhattan plot of continuous traits PheWAS association with IL27.

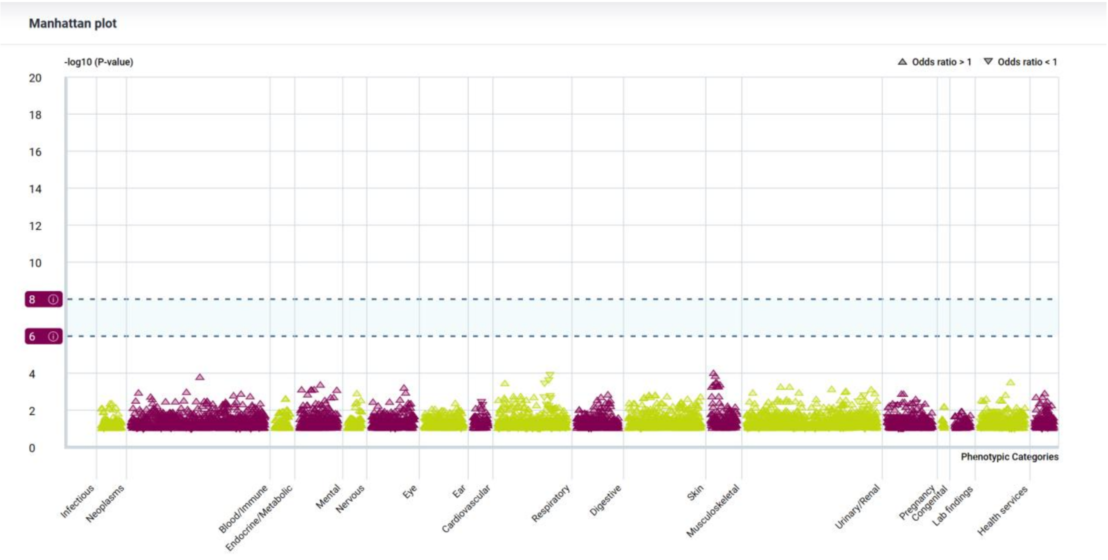

Figure S52. The manhattan plot of binary traits PheWAS association with MAPK3.

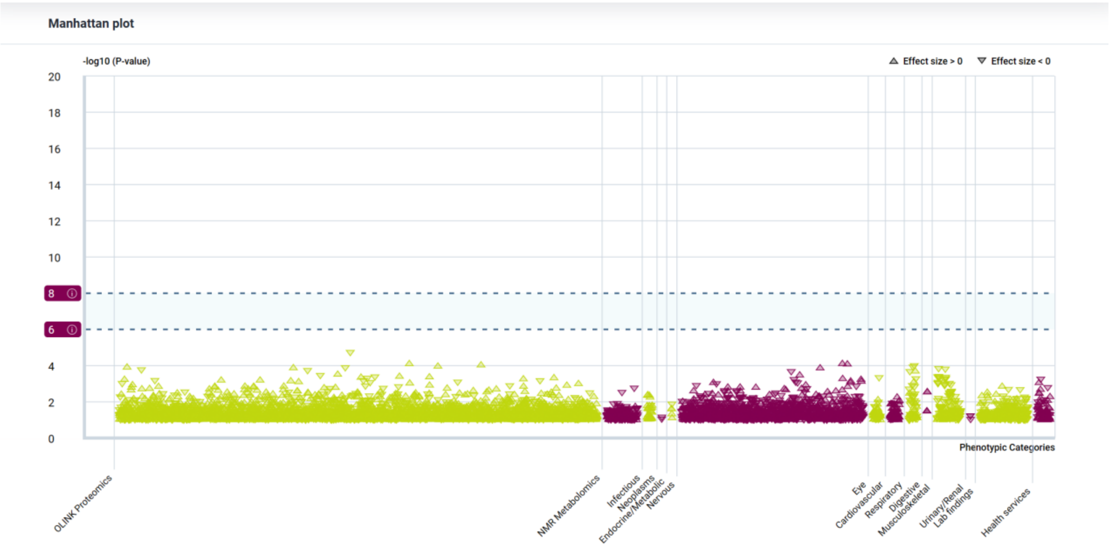

Figure S53. The manhattan plot of continuous traits PheWAS association with MAPK3.

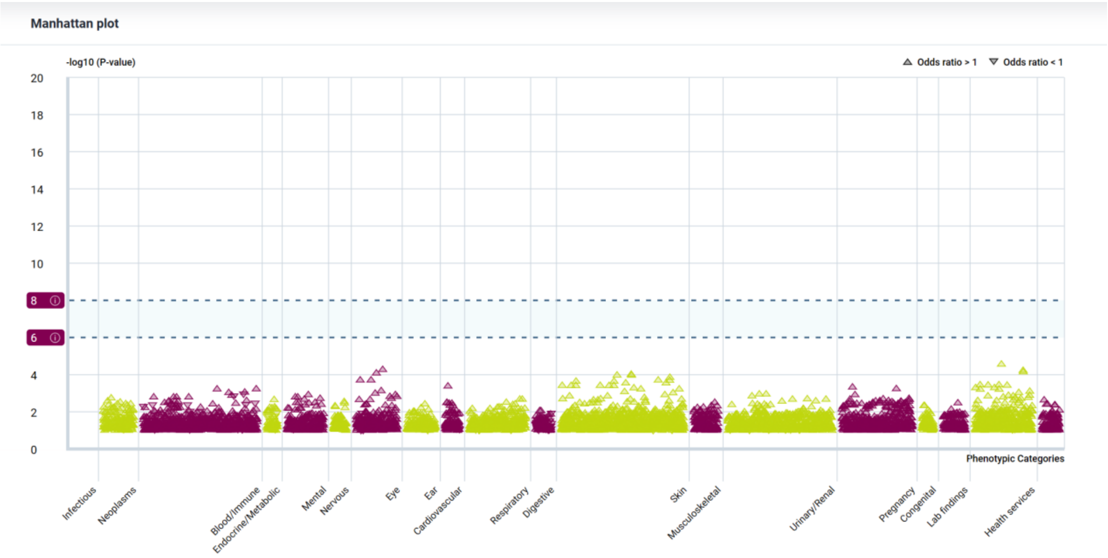

Figure S54. The manhattan plot of binary traits PheWAS association with NRBP1.

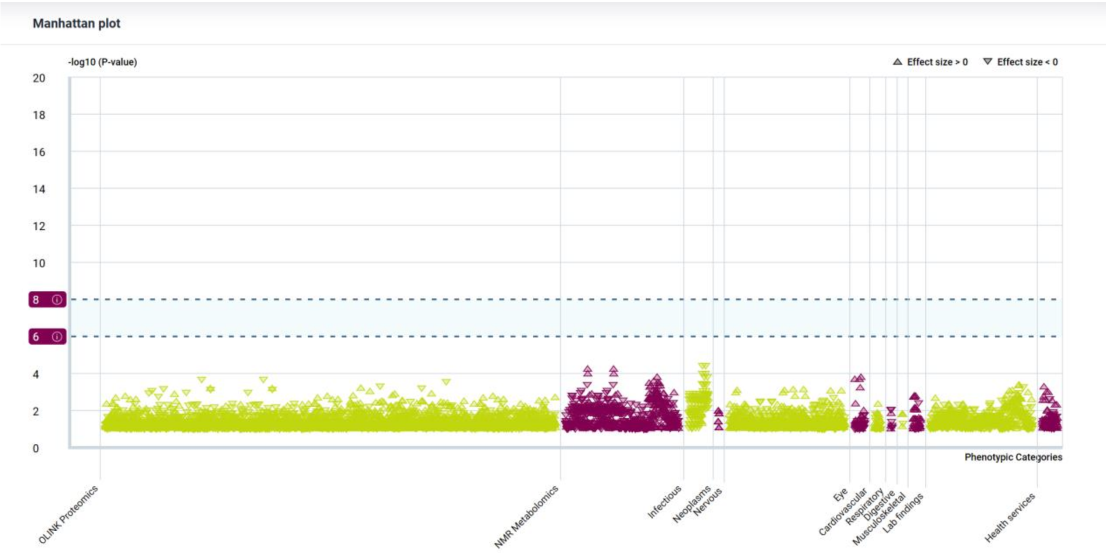

Figure S55. The manhattan plot of continuous traits PheWAS association with NRBP1.
